# Genomic Epidemiology and Phylodynamic Analysis of Enterovirus A71 Reveal Its Transmission Dynamics in Asia

**DOI:** 10.1128/spectrum.01958-22

**Published:** 2022-10-06

**Authors:** Jinbo Xiao, Keqiang Huang, Huanhuan Lu, Yang Song, Zhenzhi Han, Man Zhang, Jichen Li, Xiaofang Zhou, Jianhua Chen, Qiuli Yu, Ming Yang, Dongmei Yan, Tianjiao Ji, Qian Yang, Shuangli Zhu, Wenbo Xu, Yong Zhang

**Affiliations:** a WHO WPRO Regional Polio Reference Laboratory and National Health Commission Key Laboratory of biosafety, National Institute for Viral Disease Control and Prevention, Chinese Center for Disease Control and Prevention, Beijing, People’s Republic of China; b Shandong Center for Disease Control and Prevention, Jinan, Shandong, People’s Republic of China; c Yunnan Center for Disease Control and Prevention, Kunming, Yunnan, People’s Republic of China; d Gansu Center for Disease Control and Prevention, Lanzhou, Gansu, People’s Republic of China; e Hebei Center for Disease Control and Prevention, Shijiazhuang, Hebei, People’s Republic of China; f Heilongjiang Center for Disease Control and Prevention, Harbin, Heilongjiang, People’s Republic of China; g Center for Biosafety Mega-Science, Chinese Academy of Sciences, Wuhan, Hubei, People’s Republic of China; Emory University School of Medicine

**Keywords:** Bayesian phylodynamics, EV-A71, hand foot and mouth disease, phylogenetics, recombination

## Abstract

Enterovirus A71 (EV-A71) is one of the main pathogens causing hand, foot, and mouth disease (HFMD) outbreaks in Asian children under 5 years of age. In severe cases, it can cause neurological complications and be life-threatening. In this study, 200 newly sequenced EV-A71 whole-genome sequences were combined with 772 EV-A71 sequences from GenBank for large-scale analysis to investigate global EV-A71 epidemiology, phylogeny, and Bayesian phylodynamic characteristics. Based on the phylogenetic analysis of the EV-A71 *3D^pol^* region, six new evolutionary lineages (lineages B, J, K, O, P, and Q) were found in this study, and the number of evolutionary lineages was expanded from 11 to 17. Temporal dynamics and recombination breakpoint analyses based on genotype C revealed that recombination of nonstructural protein-coding regions, including *3D^pol^*, is an important reason for the emergence of new lineages. The EV-A71 epidemic in the Asia-Pacific region is complex, and phylogeographic analysis found that Vietnam played a key role in the spread of subgenotypes B5 and C4. The origin of EV-A71 subgenotype C4 in China is East China, which is closely related to the prevalence of subgenotype C4 in the south and throughout China. Selection pressure analysis revealed that, in addition to VP1 amino acid residues VP1-98 and VP1-145, which are associated with EV-A71 pathogenicity, amino acid residues VP1-184 and VP1-249 were also positively selected, and their functions still need to be determined by biology and immunology. This study aimed to provide a solid theoretical basis for EV-A71-related disease surveillance and prevention, antiviral research, and vaccine development through a comprehensive analysis.

**IMPORTANCE** EV-A71 is one of the most important pathogens causing HFMD outbreaks; however, large-scale studies of EV-A71 genomic epidemiology are currently lacking. In this study, 200 new EV-A71 whole-genome sequences were determined. Combining these with 772 EV-A71 whole-genome sequences in the GenBank database, the evolutionary and transmission characteristics of global and Asian EV-A71 were analyzed. Six new evolutionary lineages were identified in this study. We also found that recombination in nonstructural protein-coding regions, including *3D^pol^*, is an important cause for the emergence of new lineages. The results provided a solid theoretical basis for EV-A71-related disease surveillance and prevention, antiviral research, and vaccine development.

## INTRODUCTION

Enterovirus A71 (EV-A71) (genus *Enterovirus*; family *Picornaviridae*) is one of the pathogens causing hand, foot, and mouth disease (HFMD) and, together with coxsackievirus A6 (CVA6), CVA16, and CVA10, is the main pathogen causing HFMD epidemics in Asia. It is a single-stranded, positive-sense RNA virus consisting of approximately 7,400 nucleotides (nt), including a 5′ untranslated region (UTR), short open reading frame (ORF), long ORF, and 3′ UTR. The long ORF can be cleaved into three polyprotein precursors, *P1*, *P2*, and *P3*, which are further divided into genes encoding structural proteins (*VP4*, *VP2*, *VP3*, and *VP1*) and nonstructural proteins (*2A*, *2B*, *2C*, *3A*, *3B*, *3C*, and *3D^pol^*). The short ORF, referred to as the upstream ORF (uORF) (the second ORF), has recently been discovered and encodes the protein ORF2p, involved in EV-A71 infection and replication (1, 2).

Humans are the only known natural host, and EV-A71 can cause neurological diseases, aseptic meningitis, acute flaccid paralysis (AFP), brain stem encephalitis, and death in severe cases (3, 4). Since 1969, when it was first isolated from specimens of patients with central nervous system (CNS) disease in California (5), EV-A71 has spread worldwide and caused numerous HFMD outbreaks (6–8). Recently, HFMD outbreaks have hit the Asia-Pacific region the hardest, becoming a major public health problem (9). The World Health Organization (WHO) Western Pacific Regional Office produced *Western Pacific Region Hand*, *Foot and Mouth Disease Surveillance Summary* biweekly to report biweekly case numbers from China, Japan, the Republic of Korea, Hong Kong SAR, China, Macau SAR, China, Singapore, and Vietnam (https://iris.wpro.who.int/handle/10665.1/10963). This has been replaced by the WHO regional event-based surveillance system.

In 1999, EV-A71 was divided into three genotypes (A, B, and C) and four subgenotypes (B1, B2, C1, and C2), according to the *VP1* region. New genotypes and subgenotypes were reported in the following decade, HFMD incidence increased, and the accompanying clinical symptoms became more severe (10–12). Some of the newly discovered genotypes are confined to specific countries and regions, such as genotype D, confined to India, and genotypes E and F, confined to parts of Africa, and are rarely identified in disease outbreaks (11, 12). Genotype A gradually disappeared during the epidemic. At present, the prevalent genotypes, B and C, contain multiple subgenotypes, some of which are closely related to HFMD outbreaks and have distinct geographical transmission characteristics.

Cocirculation of subgenotypes B5 and C4 is the main feature of the current EV-A71 epidemic. Subgenotype B5 was first discovered in Malaysia in 1999 and then separately in Thailand, China, Vietnam, and other neighboring countries, with a stable circular spread in Asia (13–17). Severe HFMD outbreaks in Japan, Singapore, and Taiwan, China, have also been associated with subgenotype B5 (18–20). In 2007, Denmark reported the first case of subgenotype B5 infection in Europe. Subsequently, B5 was also detected in France, which was analyzed and found to have high sequence homology with Asian sequences and was more likely to be imported from Asia (13, 21). The subgenotype C4 was first identified in China in 2004 and was closely associated with severe HFMD cases. Some studies have found that C4 can cause more severe HFMD symptoms than B5 (22). C4 is the only transmission chain with a long-standing epidemic in mainland China, and its important evolutionary branch, C4a, is thought to be closely linked to the massive HFMD outbreaks in China (23). Currently, the branch has spread to Denmark, Vietnam, Cambodia, and other countries (22, 24, 25). China and Southeast Asia are the main regions of endemicity for subgenotypes B5 and C4, respectively. Clarifying the transmission characteristics could suggest the tracing and control of EV-A71, but studies on this subject are still lacking.

Recombination plays an important role in the evolution and genetic variation of enteroviruses. Recombination events were readily found in many new enterovirus genomes (26, 27). Such events may change the pathogenicity of the virus by affecting the tissue tropism of the virus in the human body (28) and are an important means of virus survival (29). Compared with other regions, enterovirus *3D^pol^* is more susceptible to recombination. It encodes RNA-dependent RNA polymerases (RdRps) with low fidelity, leading to misincorporations during viral replication, and recombination may be used to reduce the accumulation of such errors in the viral genome (28, 30, 31). Because of the importance of *3D^pol^* in the evolution of enteroviruses, the recombination forms (RFs) of enterovirus *3D^pol^* according to their phylogenetic characteristics and nucleotide differences have been classified by scholars. EV-A71 was classified as having 11 RFs (32), CVA6 as having 13 RFs (33), and ECHO30 as having 38 RFs (34). With the widespread prevalence of EV-A71 and the continued occurrence of recombinant events, research on *3D^pol^* should continue to progress.

A well-established data set can more accurately restore the process of viral evolution and transmission. In this study, 200 newly sequenced EV-A71 whole genomes (covering 24 provinces in mainland China) were combined with sequences available from GenBank to form a large EV-A71 genome-wide data set, which was used as the basis for the study of EV-A71 recombination diversity, epidemiological characteristics, phylogenetic characteristics, and Bayesian phylodynamics. The results reveal the latest recombination characteristics of EV-A71 and the process of epidemic transmission in China and Southeast Asia and are important for disease surveillance, prevention, and control and antiviral research.

## RESULTS

### Phylogeny of EV-A71 VP1 and *3D^pol^*.

A total of 259 whole-genome sequences of EV-A71 from 19 countries were screened for phylogenetic analysis of *VP1* and *3D^pol^* (Table 1; also, see Table S3 in the supplemental material), with 121 newly sequenced sequences and 138 sequences already present in GenBank (including the prototype strain U22521/BrCr/USA/1970, referred to here as strain U22521). Using the genome of strain U22521 as the standard for genome division, 259 sequences were split into four large data sets for the ORF, *P1*, *P2*, and *P3*, and the mean nucleotide distances between sequences were 13.8%, 11.7%, 14.3%, and 16.0%, respectively. The sequences were further split into 10 small data sets for *VP4*, *VP2*, *VP3*, *VP1*, *2A*, *2B*, *2C*, *3AB*, *3C*, and *3D^pol^*, and the mean nucleotide distances between the sequences were 11.3%, 11.7%, 12.5%, 11.3%, 13.4%, 16.2%, 14.1%, 16.0%, 15.8%, and 16.1%, respectively. The difference in the nonstructural-protein-coding region was higher than that in the structural-protein-coding region, and the differences in *VP1* and *3D^pol^* in the phylogenetic tree were consistent with this result.

**TABLE 1:** Global EV-A71 evolutionary lineages

Lineage	Genotype or subgenotype(s)	No.^*a*^	Divergence^*b*^	Country(ies)/region(s) of isolation	Yr(s) isolated
A	A	1	NA	United States	1970
B	B0	2	0.003	Netherlands	1966
C	B1	2	0.020	Taiwan (China)	1986
D	B2	2	0.004	Singapore, United States	1987
E	C1, C2	19	0.089	Netherlands, Taiwan (China), Malaysia, Australia, Singapore, Canada, Thailand, United States, Cameroon	1991–2016
F	B3	4	0.008	Malaysia, Singapore, Australia	1997–1999
G	C3	2	0.009	Korea	2000
H	B4, B5	28	0.057	Malaysia, Taiwan (China), Singapore, Thailand, Vietnam, Cambodia	2001–2015
I	C4	173	0.052	Cambodia, mainland China, Taiwan (China), Canada, Vietnam, Laos	2001–2019
J	F	2	0.000	Madagascar, Cameroon	2004, 2008
K	C4	2	0.053	Thailand	2006, 2008
L	C5	7	0.032	Taiwan (China), Vietnam	2006–2012
M	C2	3	0.002	Taiwan (China)	2008
N	C4	1	NA	Mainland China	2008
O	C4	2	0.028	Mainland China	2011
P	E	1	NA	Niger	2013
Q	C1	8	0.015	Germany, United States, Japan, Denmark	2015–2017

a Number of sequences analyzed in each lineage.

b Overall mean distances. Values were calculated from the complete *3D^pol^* region of lineages. NA, the value was not calculated when there is only one sequence in lineage.

**FIG 1:**
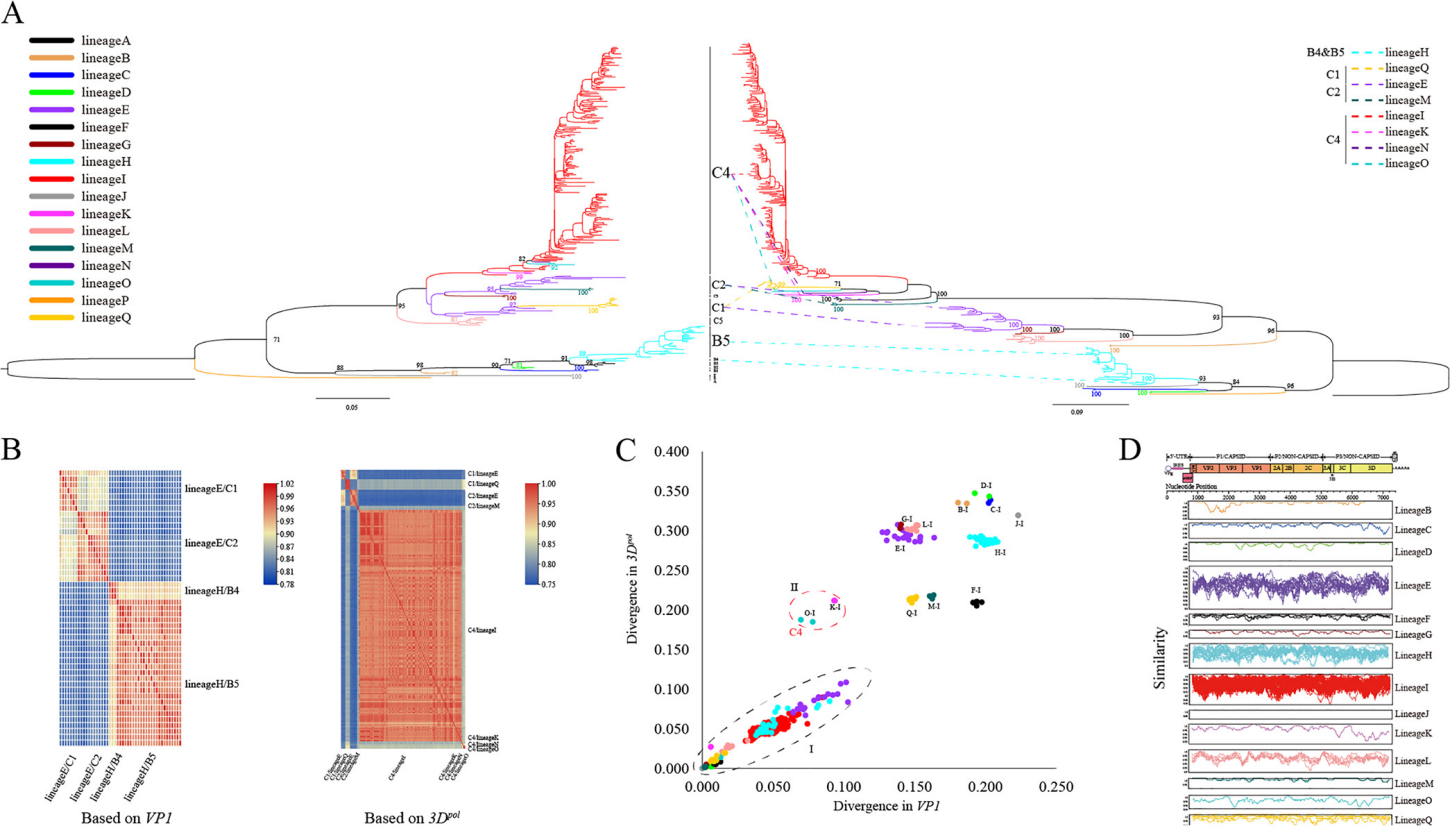
Phylogenetic analysis of 259 EV-A71. (A) (Left) ML tree constructed from the *VP1* region of 259 sequences with the corresponding genotype/subgenotype of each clade labeled; (right) ML tree constructed from the *3D^pol^* of 259 sequences. Different colors are used to distinguish the different lineages, and the two ML trees are colored according to the lineage to which the sequences belong. Dotted lines indicate subgenotypes and lineages with phylogenetic differences. (B) (Left) Heat map of the nucleotide similarity of the lineage E and H *VP1* region sequences, where the two subgenotypes belonging to the same lineage have reduced similarity in the *VP1* region, and different lineages have an even lower similarity; (right) heat map of nucleotide similarity of the C1, C2, and C4 *3D^pol^* regions. Again, the similarity of different lineages belonging to the same subgenotype significantly decreases. (C) Pairwise distance comparison of *VP1* and *3D^pol^* sequences. The area labeled “I” shows the comparison of intralineage sequences, and the rest compares different lineages with lineage I. The area labeled “II” compares lineages with lineage I of the same C4. The colors of different lineages are as in panel A. (D) Comparison of intralineage sequence similarity based on ORF region using sliding-window nucleotide similarity analysis, with 200 nucleotide windows moving in steps of 20 nucleotides. A lineage containing only 1 sequence was not analyzed.

According to the *3D^pol^* phylogenetic tree, global EV-A71 can be divided into 17 evolutionary lineages: A, B, C, D, E, F, G, H, I, J, K, L, M, N, O, P, and Q. Lineage A contains only the prototype strain U22521, and lineages N and P contain only one sequence. Lineages E, H, and I contained the most sequences, and L and Q also contained more sequences, forming the main evolutionary lineages, and other lineages contained sporadic sequences (>1). Evident differences were observed in topologies when the phylogenetic trees formed by analyzing *VP1* and *3D^pol^* were compared (Fig. 1A). The main difference is that some subgenotypes form multiple lineages; for example, C1 forms lineages E and Q, C2 forms lineages E and M, and C4 forms lineages I, K, N, and O. Some subgenotypes merge to form the same lineage; B4 and B5 form lineage H, and C1 and C2 form lineage E. These results were confirmed by the heat map of nucleotide similarity between the corresponding sequences, *VP1* and *3D^pol^*, with low *VP1* nucleotide similarity in different subgenotypes of the same lineage and low *3D^pol^* nucleotide similarity in different lineages of the same subgenotype (Fig. 1B).

Comparisons of pairwise distances also showed significant differences between lineages in the *VP1* and *3D^pol^* sequences (Fig. 1C). The intralineage sequences showed a positive linear correlation between *VP1* and *3D^pol^*. However, the difference between lineages was noticeable. With regard to lineage I and other lineages, for example, lineages O and K also belong to C4; their divergence with lineage I on *VP1* is small, but *3D^pol^* divergence is evident. Most lineages differed significantly from lineage I in terms of both *VP1* and *3D^pol^*. Overall, the difference between the different lineages in *3D^pol^* was markedly higher than that in *VP1*.

A sliding-window nucleotide analysis of intralineage ORF sequences was performed, which showed high homology (Fig. 1D). Furthermore, in the analysis of the phylogenetic tree based on ORF sequences (Fig. S4), the phylogenetic characteristics of the *3D^pol^* region were closer to the ORF than to the *VP1* region, and the clades divided by *3D^pol^* were more similar to the maximum-likelihood (ML) trees of the ORF.

### Evolutionary dynamics and recombination breakpoint analysis of global EV-A71.

**FIG 2:**
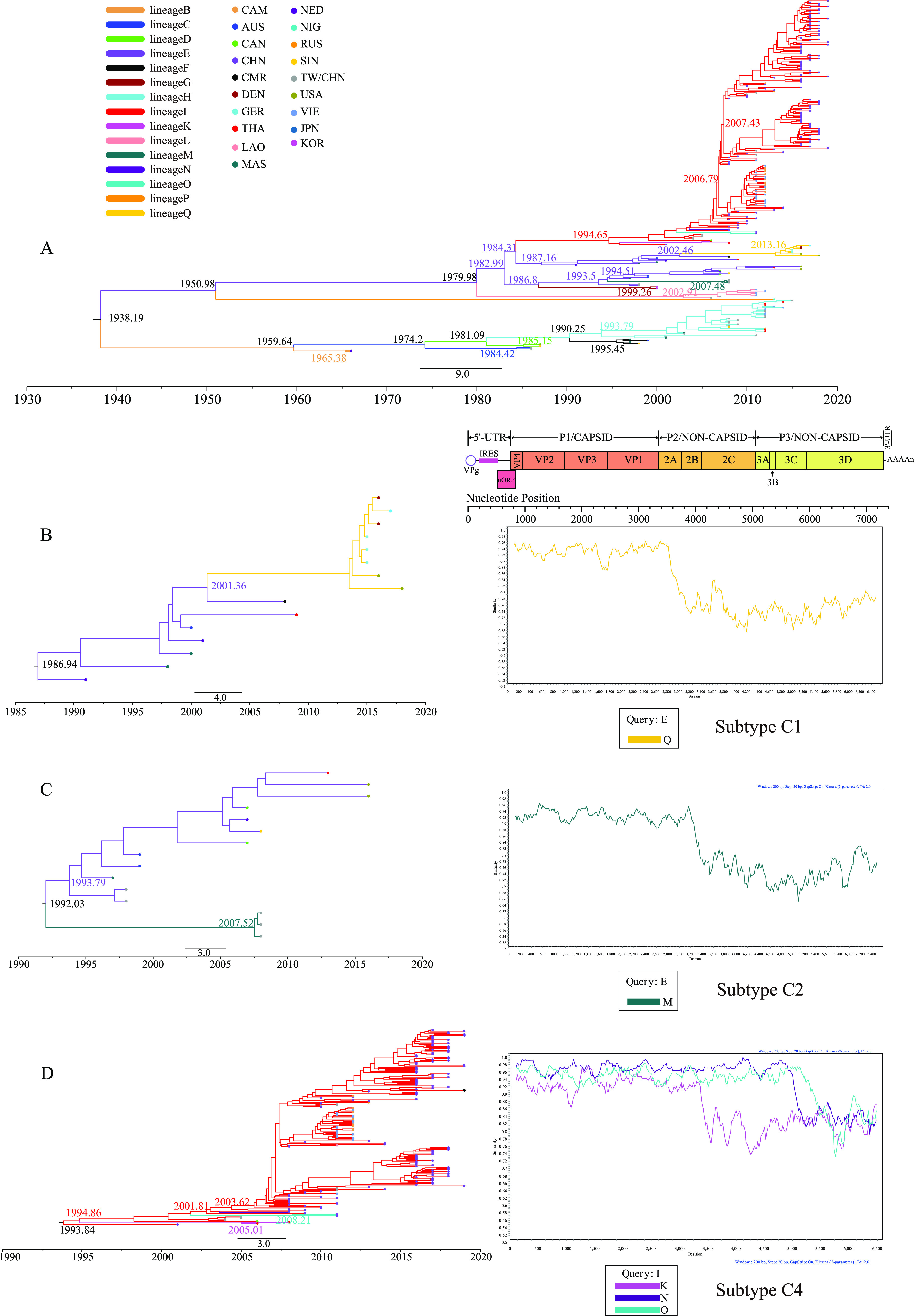
Temporal dynamics analysis of global EV-A71. (A) A maximum clade credibility (MCC) tree was constructed based on 256 EV-A71 *VP1* sequences, with the branches colored according to lineage and the tip colored according to region. (B to D) Temporal dynamics analysis of subgenotypes (left) and recombination breakpoint analysis (right) of subgenotypes C1, C2, and C4, respectively. The colors of the MCC tree are consistent with those in panel, and the query groups are lineage E, lineage E, and lineage I, respectively.

The best model combination for the 256 EV-A71 evolutionary dynamics analyses was the uncorrelated relaxed clock (lognormal) and constant size (35). The maximum clade credibility (MCC) tree based on entire *VP1* region showed that around 1938, EV-A71 began to form two major genotypes, B and C, which evolved into two independent branches (Fig. 2A). Genotype B began to evolve into subgenotypes in 1959, and each new subgenotype emerged with a new lineage, except B4 and B5, which shared lineage H. The evolution of the subgenotypes showed consecutive substitutions: B1 (lineage C) replaced B0 (lineage-B), B2 (lineage D) replaced B1 (lineage C), and B3 (lineage F) replaced B2 (lineage D), and two subgenotypes (lineages) in a substitution relationship shared the same time to the most recent common ancestor (tMRCA). These subgenotypes occurred sporadically, with different modes of recombination present in *3D^pol^* and confined to certain countries: B0 was confined to the Netherlands, and B1 mainly occurred in Taiwan, China. The subgenotypes B4 and B5 began to appear in 1990 and 1993, respectively, and the two subgenotypes coexisted for decades, consistently sharing lineage H without recombination events, leading to the creation of new lineages. B5 (lineage H) has gradually become dominant in transmission, leading to many outbreaks in the last 3 decades, including the 2007–2009 and 2011–2012 HFMD outbreaks in Taiwan (China) and mainland China and the 2011–2014 HFMD outbreaks in Thailand and Vietnam.

Genotype C began to form two branches in 1979, one forming C5 with a more stable *3D^pol^* sequence, which existed as lineage L during the epidemic. The other branch was composed of three separate branches in 1982. The first isolate was the subgenotype C1, which spread as an independent branch for 27 years (up to 2018) and experienced a recombination event between 2001 and 2015 when the evolutionary lineage shifted from lineage E, which had been continuously prevalent for 18 years, to lineage Q. Analysis of recombination breakpoints revealed apparent differences between the two lineages from the beginning of the *2A* region of the sequence (Fig. 2B). C2 and C3 share the same branches. C3 formed a small cluster that underwent recombination events from 1986 to 1999 to form lineage G. C2 first spread as lineage E and was endemic for 19 years as of 2016. Between 1992 and 2008, C2 recombined to create a new lineage, M, which was coprevalent with lineage E. The differences between the two lineages began to increase in the *2B* region (Fig. 2C). C4, a major subgenotype of EV-A71, has also undergone several recombination events during the epidemic, but lineage I has been dominant. Lineage K, isolated in Thailand in 2006 and 2008, lineage N, isolated in China in 2008, and lineage O, isolated in China in 2011, all existed as disseminated lineages. Recombination breakpoint analysis with lineage I as the query group revealed that the sequence similarity of lineages K and I started to decrease at *2C*, and the sequence similarity of both lineages N and O and lineage I started to decrease in the *3C* region.

The substitution rate for EV-A71 was 3.79 × 10^−3^ substitution/site/year (high-probability distribution [HPD] range, 3.29 × 10^−3^ to 4.29 × 10^−3^) (Table 2), and tMRCA was 81.88 (HPD range, 66.92 to 99.59). The substitution rate for each genotype and subgenotype was 4.10 × 10^−3^ to 4.85 × 10^−3^ substitution/site/year. The three largest evolutionary lineages, lineages E, H, and I in *3D^pol^*, had a substitution rate very close to that of *VP1*, and the tMRCA was somewhat similar. This is consistent with the fact that the divergence of the three lineages in the *VP1* and *3D^pol^* regions showed a significant positive correlation (Fig. 1C).

**TABLE 2:** Basic parameters of data sets and the results of the Markov chain Monte Carlo (MCMC) analysis

Genotype or lineage	No.^*a*^	Divergence^*b*^		*dN*/*dS*^*c*^		MCMC (BEAST)			
						Substitution rate (10^−3^) (95% HPD)		tMRCA (95% HPD)	
		*VP1*	*3D^pol^*	*VP1*	*3D^pol^*	*VP1*	*3D^pol^*	*VP1*	*3D^pol^*
All	256	0.11	NA^*d*^	0.03	NA	3.79 (3.29–4.29)	NA	81.88 (66.92–99.59)	NA
Individual genotypes									
B	38	0.08	NA	0.05	NA	4.10 (3.11–5.11)	NA	56.12 (49.20–64.17)	NA
C	217	0.08	NA	0.03	NA	4.29 (3.72–4.87)	NA	37.23 (32.67–41.87)	NA
Individual subgenotypes									
B5	25	0.04	0.05	0.06	0.06	4.26 (3.18–5.37)	4.48 (3.25–5.57)	14.36 (13.06–15.90)	14.56 (13.14–16.47)
C1	15	0.07	NA	0.02	NA	4.15 (3.14–5.20)	NA	31.28 (28.54–34.13)	NA
C2	15	0.07	NA	0.02	NA	4.85 (3.60–6.19)	NA	24.05 (20.90–28.28)	NA
C4	178	0.05	NA	0.04	NA	4.42 (3.79–5.03)	NA	25.11 (22.01–28.42)	NA
Individual lineages									
E	19	0.09	0.09	0.01	0.04	4.29 (3.21–5.42)	4.06 (3.27–4.78)	34.38 (28.08–41.16)	34.57 (30.53–38.76)
H	28	0.05	0.06	0.05	0.06	3.62 (2.64–4.62)	3.36 (2.33–4.53)	24.68 (19.07–31.04)	25.04 (18.89–32.15)
I	173	0.05	0.05	0.04	0.07	4.45 (3.81–5.05)	4.61 (4.10–5.13)	25.06 (21.89–28.65)	23.72 (21.22–26.70)

a Number of sequences analyzed in each data set.

b Overall mean distance.

c *dN*/*dS* for each data set obtained by using the SLAC method.

d NA, the values of the *3D^pol^* region were not calculated, because *3D^pol^* sequences in the data set were not monophyletic.

### Phylogeographic analysis of Asia subgenotypes B5 and C4.

Upon phylogeny-trait association analysis of subgenotype B5, statistical tests for association index (AI), parsimony score (PS), and maximum monophyletic clade (MC) values showed that all were significant (*P < *0.05) (Table 3), indicating that subgenotype B5 has significant spatial structure and more localized evolution in these regions. The analysis of subgenotype C4 also showed that the sequence had a significant spatial structure in the regions to which it belonged (Table S12).

**TABLE 3:** Analysis of the spatial structure of B5 strains in Asia^*a*^

Statistic	Mean (95% HPD CI)		*P*
	Observed	Null	
AI	0.59 (0.41–0.84)	6.49 (5.64–7.41)	<0.001
PS	6.79 (6.00–8.00)	39.61 (36.26–43.08)	<0.001
MC (Thailand)	12.99 (13.00–13.00)	2.62 (2.01–3.95)	0.01
MC (Taiwan [China])	16.98 (17.00–17.00)	2.83 (2.05–4.11)	0.01
MC (Vietnam)	15.74 (8.00–19.00)	2.10 (1.35–3.17)	0.01

a HPD CI, highest posterior density confidence interval; AI, association index; PS, parsimony score; MC, maximum monophyletic clade.

**FIG 3:**
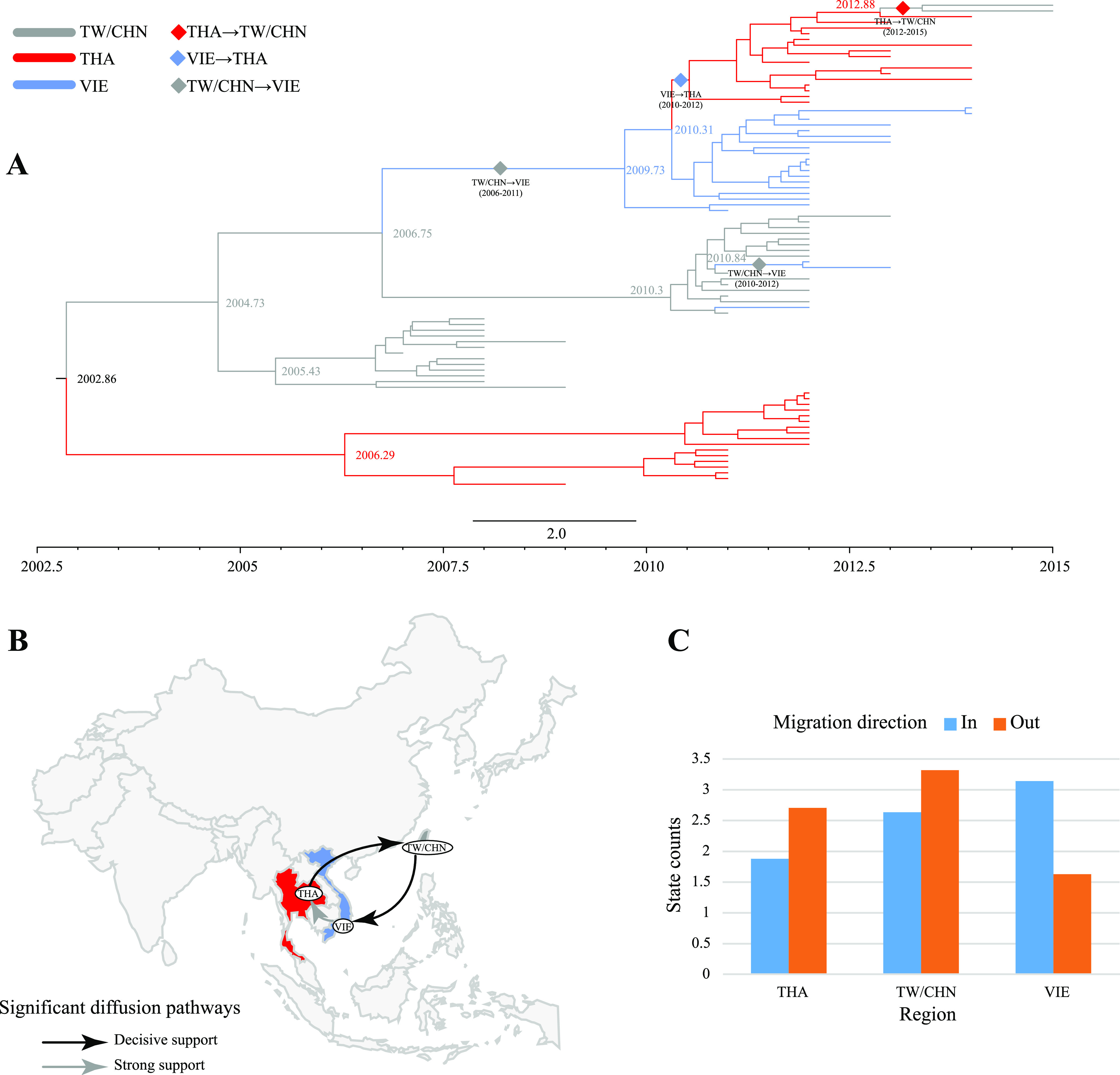
Phylogeographic analysis of B5 in Asia. (A) MCC tree constructed based on 85 B5 *VP1* sequences. Branch colors indicate inferred location states, diamonds indicate the presence of B5 spread between regions, and the spread direction and time range are marked at the corresponding positions. (B) B5 spatial diffusion pathways in Taiwan (China), Vietnam, and Thailand. Only migration pathways with a mean indicator of >0.5 are shown. Solid black arrows indicate migration pathways with decisive support (BF > 1,000), dashed black arrows indicate migration pathways with very strong support (150 < BF < 1,000), solid gray arrows indicate migration pathways with strong support (20 < BF < 150), and dashed gray arrows indicate migration pathways with support (3 < BF < 20). All migration pathways in this paper follow this criterion. (C) Histogram of the total number of location state transitions inferred from the three regions.

B5 circulated in Thailand, Taiwan, and Vietnam, with the highest mean migration rate from Taiwan, China, to Vietnam, followed by Thailand to Taiwan, China, and the lowest mean migration rate from Vietnam to Thailand (Table S13). Based on the MCC tree results, the first transmission of B5 from Taiwan, China, to Vietnam occurred from 2006 to 2011, and the second was from 2010 to 2012 (Fig. 3A). The Vietnam transmission to Thailand took place between 2010 and 2012. There was a small amount of transmission from Thailand to Taiwan, China, between 2012 and 2015. The inference of phylogeographic analysis of the migration direction of B5 confirmed the results of MCC (Fig. 3B), and the results were supported by a high Bayes factor (BF) and posterior probability values (Table S13). The state counts inferred by the Markov jumps method indicate that Taiwan, China, dominates out-migration, followed by Thailand and Vietnam (Fig. 3C). Vietnam became the main in-migration area for B5, followed by Taiwan (China) and Thailand. This result is consistent with the inference of migration routes, with inputs and outputs of B5 at all three areas.

**FIG 4:**
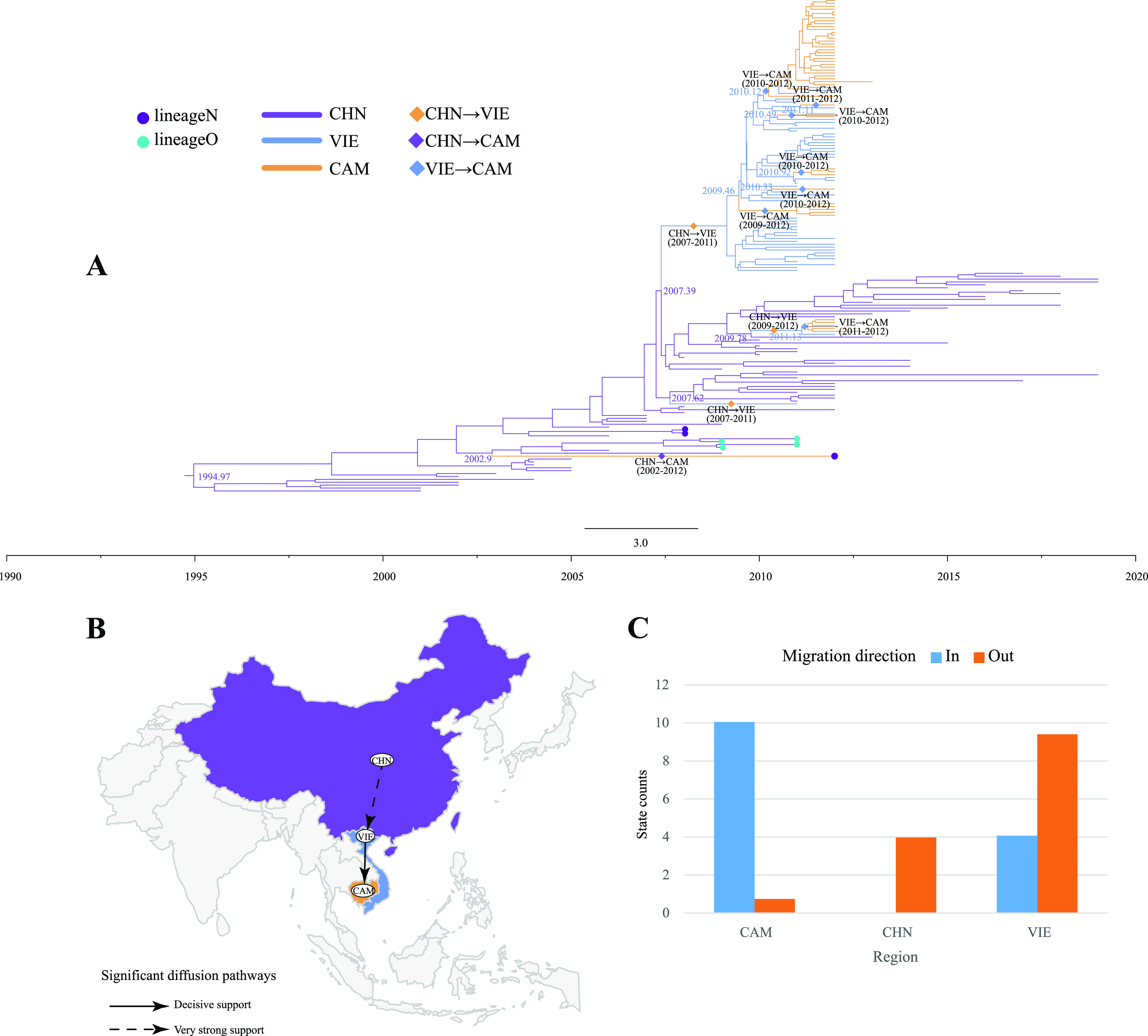
Phylogeographic analysis of C4 in Asia. (A) MCC tree constructed based on 170 C4 *VP1* sequences. Branch colors indicate inferred location states. Lineages N and O are highlighted by dots on the tip. Diamonds indicate the existence of C4 spread between regions, with the direction and time range of spread noted close to them. (B) Spatial diffusion pathways of C4 in China, Vietnam, and Cambodia, showing only migration pathways with BF values of >3 and indicators of >0.5. (C) Histogram of the total number of location state transitions inferred from the three regions.

C4 was transmitted among China, Vietnam, and Cambodia, with a high mean rate of migration from Vietnam to Cambodia. China to Vietnam was also supported by the mean migration rate (Table S14). Lineage I of C4 recombined during transmission in China to produce lineages N and O on the MCC tree. A sporadic propagation event occurred from 2002 to 2012, and lineage N was transmitted from China to Cambodia. Subgenotype C4 was transmitted from China to Vietnam three times, twice between 2007 and 2011 and once between 2009 and 2012 (Fig. 4A). There were several C4 migrations between Vietnam and Cambodia, mainly from Vietnam to Cambodia. The phylogeographic analysis inferred two C4 transmission routes (Fig. 4B), one from China to Vietnam and the other from Vietnam to Cambodia, confirmed by the BF and posterior probability values (Table S14). This conclusion is also well supported by the observed state changes, with much greater out-migration from Vietnam and China than from Cambodia and with Cambodia becoming the most dominant in-migration region for C4 (Fig. 4C).

### Phylogeographic analysis of China C4.

According to the phylogeny-trait association analysis results (Table S15), the statistical tests for AI and PS values showed significant levels, and some MCs were statistically insignificant (*P* > 0.05), indicating the existence of geographic mixing in China for C4.

**FIG 5:**
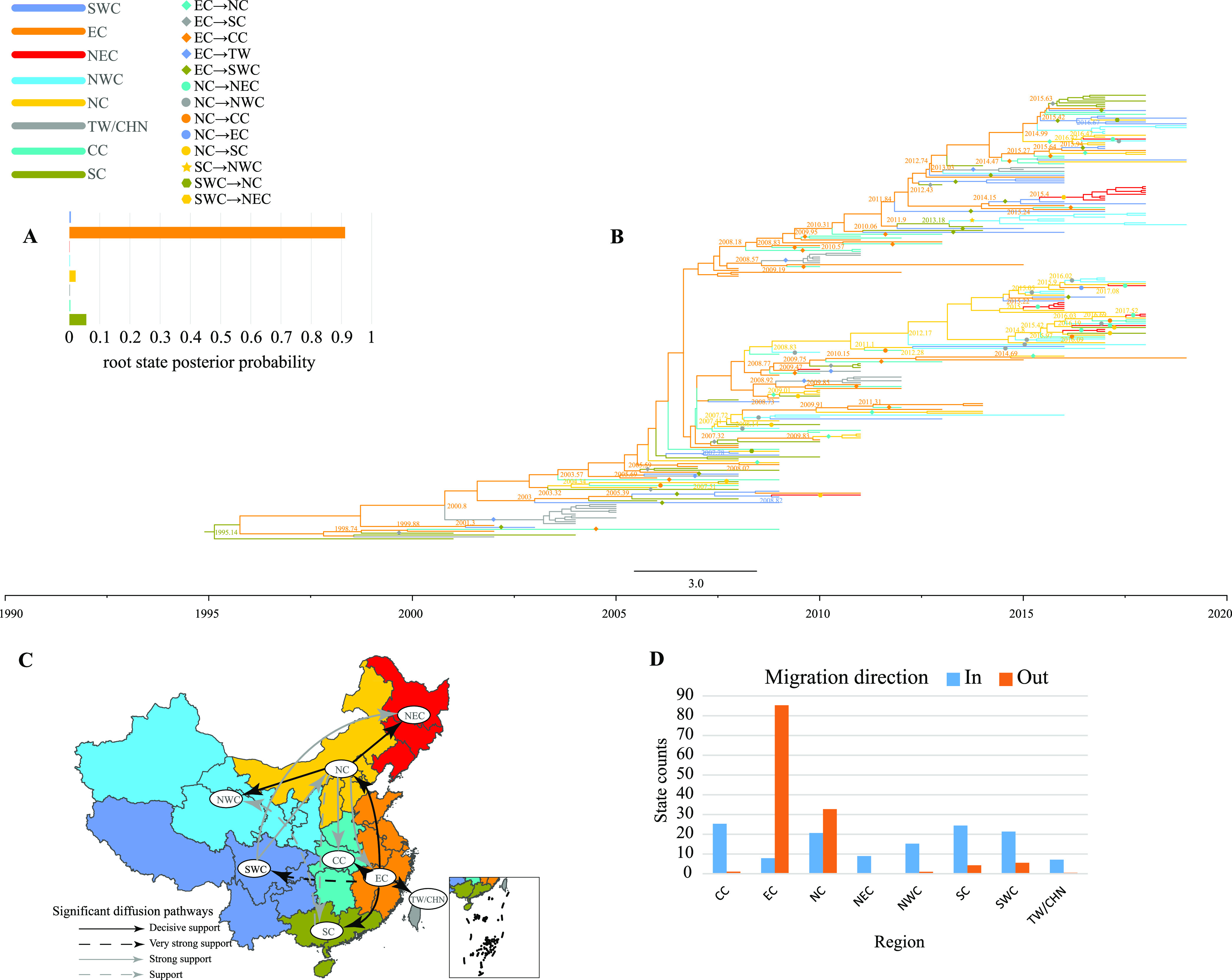
Phylogeographic analysis of C4 in China. (A) Histogram of root state posterior probabilities in eight regions of China. Different colors represent different regions. The horizontal coordinate shows posterior probabilities, and the vertical coordinate shows regional distributions. (B) MCC tree constructed based on 234 *VP1* sequences; colors of branches indicate inferred location states. Solid shapes represent the propagation of C4 between different regions. (C) Spatial diffusion pathways of C4 in eight regions in China. Only migration pathways with BF values of >3 and indicators of >0.5 are shown. (D) Histogram of the total number of inferred location state transitions for the eight regions.

The root state posterior probabilities reflected that East China was the origin of C4 (Fig. 5A). As observed from the MCC tree (Fig. 5B), C4 spread mainly from East China and North China to other regions, and this situation existed in different years, leading to a gradual increase in the number of C4 isolates in other regions; the higher mean migration rate supported the MCC tree results (Table S16). In addition, sporadic transmission events have occurred in other regions. Phylogeographic analysis revealed six decisive migration pathways (Fig. 5C) and several pathways supported by high BF and posterior probability values (Table S16). It also confirmed the important role of East China and North China in the spread of the epidemic, with the decisive migration pathway of East China covering almost all of southern China. However, North China dominated the epidemic in northern China. The state count results provided evidence for the previous inference that East China and North China were the major out-migration areas, with C4 in-migration in all areas (Fig. 5D).

### EV-A71 selective pressure analysis.

**FIG 6:**
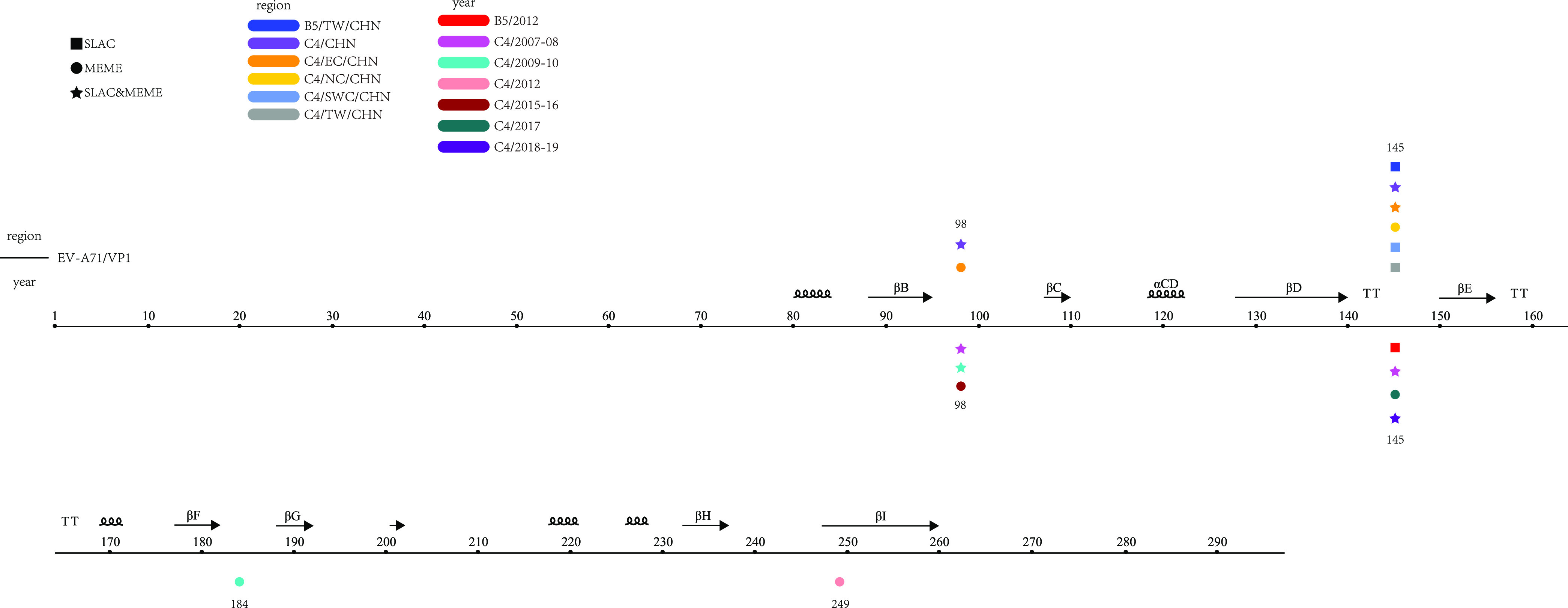
Selective pressure analysis of EV-A71. The black line represents amino acid sequences translated by the EV-A71 *VP1* region, and data sets from different regions and times are indicated by different colors. The presence of markers directly above and below the amino acid site indicated that the site had a positive selection. Squares indicate positive selection sites supported only by SLAC, circles indicate positive selection sites supported only by MEME, and stars indicate support by both methods. The markers directly above and below sites indicate positive selection sites found in data sets from different regions and years, respectively.

The B5 and C4 *VP1* sequences were divided into multiple data sets according to time and region variables. The results show that each data set showed a low mean ratio of nonsynonymous to synonymous substitutions (*dN*/*dS*) (0.0192 to 0.0827) (Table 4), and most of the nucleotide substitutions were synonymous. No significant differences were observed in *dN*/*dS* ratios for *VP1* or *3D^pol^* of B4 and B5 of lineage H (Table S17). The *dN*/*dS* ratios of lineages E and Q of C1 showed more significant differences only in *VP1*, whereas lineages E and M of C2 showed significant differences in *dN*/*dS* ratios for either *VP1* or *3D^pol^*. Analysis of the *VP1* coding region revealed that multiple data sets had one or two positive selection sites in amino acid residues VP1-98 and VP1-145 (Fig. 6), located in the BC and DE loops of EV-A71. In addition, positive selection sites were also found in amino acid residues VP1-184 (FG loop) and VP1-249 (HI loop) through the C4 (2009 to 2010) and C4 (2012) data sets.

**TABLE 4:** Selective pressures in *VP1* region of Asian B5 and C4 in different regions and years

Country/region or year	Subgenotype	No.^*a*^	SLAC		MEME	
			*dN*/*dS*	Positive selection site (amino acid position)	*dN*/*dS*	Positive selection site (amino acid position)
Countries/regions						
Thailand	B5	33	0.0475	NA^*b*^	0.0424	NA
Vietnam	B5	22	0.0218	NA	0.0192	NA
Taiwan (China)	B5	77	0.0827	145	0.0729	NA
Central China	C4	46	0.0494	NA	0.0449	NA
East China	C4	153	0.0588	145	0.0531	98/145
North China	C4	73	0.0539	NA	0.0491	145
Northeast China	C4	21	0.0298	NA	0.0271	NA
Northwest China	C4	39	0.0351	NA	0.0319	NA
South China	C4	49	0.0336	NA	0.0303	NA
Southwest China	C4	63	0.0387	145	0.0349	NA
Taiwan (China)	C4	43	0.0466	145	0.0415	NA
China	C4	487	0.0587	98/145	0.0531	98/145
Cambodia	C4	50	0.0401	NA	0.0368	NA
Vietnam	C4	157	0.0479	NA	0.0440	NA
Yrs						
2003–2009	B5	33	0.0696	NA	0.0614	NA
2011	B5	11	0.0413	NA	0.0362	NA
2012	B5	70	0.0590	145	0.0521	NA
2013	B5	10	0.0520	NA	0.0463	NA
2014–2015	B5	8	0.0236	NA	0.0210	NA
2001–2006	C4	33	0.0508	NA	0.0457	NA
2007–2008	C4	64	0.0770	98/145	0.0694	98/145
2009–2010	C4	84	0.0655	98	0.0596	98/184
2011	C4	108	0.0415	NA	0.0377	NA
2012	C4	140	0.0496	NA	0.0454	249
2013–2014	C4	56	0.0524	NA	0.0477	NA
2015–2016	C4	86	0.0368	NA	0.0330	98
2017	C4	62	0.0383	NA	0.0348	145
2018–2019	C4	62	0.0405	145	0.0370	145

a Number of sequences analyzed in each data set.

b NA, No positive selection site exists.

## DISCUSSION

This is the first large-scale collection of specimens from mainland China from 2016 to 2020 and a global EV-A71 genomic study, which is an important complement to the study of EV-A71 epidemic characteristics and transmission dynamics. We obtained 200 complete EV-A71 genome sequences through sequencing, covering most provinces and cities in mainland China, particularly samples from Yunnan, Hebei, and Shandong Provinces, where EV-A71-related HFMD outbreaks frequently occur. The publication of whole-genome data has enriched the number of EV-A71 sequences in recent years, which is beneficial for the study of genetic diversity and evolution of EV-A71. However, the genome of EV-A71 is still undergoing recombination, and new recombination events usually accompany the emergence of new subgenotypes. In addition, phylogeographic analyses indicated circulating transmission of B5 and C4 among several Asian countries with high EV-A71 prevalence.

Phylogenetic analysis showed that the occurrence of recombination events led to much greater variation in *3D^pol^* than in *VP1* for different EV-A71 lineages, and the topology of the *3D^pol^* phylogenetic tree was closer to the whole genome than that of *VP1* (Fig. S4). Lineage delineation is an important tool for genome-wide studies of EV-A71. Unlike CVA16, which underwent limited recombination events (36), frequent recombination occurred in *3D^pol^* of EV-A71, and compared with the previous study (32), six new lineages were added, namely, lineages B, J, K, O, P, and Q. Lineage Q is still prevalent. Although EV-A71 frequently generates recombinants, it also shows stability. When B4 and B5 are transmitted together, their *3D^pol^* always exists as lineage H. C4 has generated four lineages, I, K, N, and O; however, lineage I has never been replaced. Recombination is an important way for viruses to evolve, but when a virus evolves to a state suitable for survival, it will be epidemic for a long time. B5 and C4 have a significant spatial structure within the main areas of epidemicity, making them more locally evolved. B5 is endemic mainly in Thailand, Vietnam, and Taiwan, China, while C4 is prevalent mainly in mainland China, indicating that EV-A71 undergoes different recombination in different regions to produce lineages adapted to the local environment and can be stably transmitted.

According to the analysis of time-related trees, there were noticeable evolutionary differences between genotypes B and C. Genotype B showed that the appearance of a new subgenotype was accompanied by the disappearance of an old subgenotype, along with the replacement of the old lineage with a new one. The recombination event did not allow B0 to B3 to adapt to the host and environment, and the epidemics were all very short-lived, failing to establish a stable lineage. B4 and B5 share lineage H and have become the only epidemic lineage of genotype B in the last 30 years, suggesting that recombination can produce lineages adapted to the environment and host, as evidenced by several B5-related HFMD outbreaks in Taiwan (China) and Southeast Asia.

Unlike genotype B, genotype C has begun to evolve simultaneously with multiple subgenotypes, C1, C2, C4, and C5, which continue to circulate to the present day. The C1- and C2-shared lineage E appeared earlier, C1 (lineage Q) and C2 (lineage M) appeared later, and lineage E spread widely, involving multiple outbreaks in Southeast Asia, and Europe (37, 38). C1 (lineage Q) was isolated mainly in European countries such as Germany, and C2 (lineage M) was confined to Taiwan, China. C4 formed four lineages during transmission; lineage I was the only one associated with HFMD outbreaks, and the rest appeared sporadically. In contrast to genotype B, recombination of genotype C produced more stable lineages that are more adaptive and widely circulating. A comparison of substitution rates in lineages E, H, and I and tMRCA in the *VP1* and *3D^pol^* regions indicates that after forming stable lineages (Table 2), *VP1* and *3D^pol^* evolved at a relatively consistent rate. The observation that nonstructural protein-coding regions, especially *3D^pol^*, played a key role in generating new lineages was verified by analysis of recombination breakpoints in subgenotypes of genotype C. The recombinantly generated lineages became prevalent after adaptation to the environment, but more like the B0- to B3-related lineages, which existed briefly and then disappeared.

HFMD is highly prevalent in the Asia-Pacific region, benefiting from the emphasis of the WHO and the establishment of HFMD surveillance networks in many countries in the region, where much of the important epidemiological information on EV-A71 is derived. B5 and C4 are currently predominant in the region and have strong geographic clustering characteristics. B5 evolved separately in Taiwan (China) and Thailand. Between 2006 and 2011, B5, which was extensively present in Taiwan, China, began to spread to Vietnam and then from Vietnam to Thailand, and after a period of circulation in Thailand, it spread to Taiwan, China, on a small scale. Since 2004, tourists from Taiwan, China, have become the leading force in Vietnam’s tourism industry. The scale of tourism from both places has been expanding, and children are the leading group of outbound travelers. This large-scale movement of people has facilitated the spread of B5. Thailand is also rich in tourism resources, which attracts people to travel and promotes the spread of the virus. C4 did not circulate through several regions like B5; it first spread mainly from China to Vietnam and then from Vietnam to Cambodia. Notably, between 2002 and 2012, lineage N was introduced into Cambodia from China. Although this is a sporadic transmission event and lineage N is not a major lineage, it indicates that lineage N is highly adaptable to the environment and has some transmission power. Vietnam is located in eastern Southeast Asia, with a long and narrow topography, and is the intersection of Southeast Asia and East Asia. It is an important juncture for human exchange, tourism, and trade between the two regions and has a humid climate. This geographical location may be responsible for the high incidence of HFMD associated with B5 and C4 in Vietnam and in other regions.

Owing to the vast population base in China, 10 million infants are born every year, and children under 5 years are a high-incidence group for HFMD, with C4-associated HFMD outbreaks occurring almost every year. The phylogeographic results revealed that C4 is highly mobile in eight regions of China, and the high posterior probability suggests that C4 originated in East China and was then transmitted to multiple regions. Secondary transmission occurred in several regions, and this flow was continuous. Throughout the epidemic, the virus spread from East China to southern China. East China contains many large cities, well-developed transportation, high population mobility, and a climate suitable for the survival of the virus, which are probably the main reasons why it has become the source of C4. C4 reached North China, and a new round of major spreading took place with it as the center, spreading to cover the northern region. As with East China, the megacity of Beijing in North China and the migratory population intensified the spread of the virus, and subgenotype C4 covered China in just over a decade.

Protection of susceptible populations through vaccination is an effective measure to prevent and control infectious disease. In general, the virus strain used for a vaccine in a given country tends to belong to the genotype circulating in that country. Since 2007, genotype C4a has been the predominant genotype circulating in mainland China, and the only vaccine strains used in three EV-A71 inactivated vaccines approved in mainland China (Sinopharm, Sinovac, and Institute of Medical Biology) all belong to the C4a genotype (39, 40). Although viruses of the same serotype but different genotypes have cross-protection (41), the best protective effect is achieved with a vaccine strain belonging to the same genotype as the circulating virus. However, if the pathogen spectrum of HFMD changes greatly, it is necessary to develop enterovirus vaccines of other serotypes.

Natural selection is another important factor driving viral evolution, and selection pressure analysis is essential for understanding viral evolution. One or two positive selection sites at *VP1* amino acid residues VP1-98 and VP1-145 of the EV-A71 were detected by multiple data sets. VP1-145 is a surface-exposed residue that maps to the 5-fold vertex and is a crucial site for binding the Fab fragment of the monoclonal antibody MA28-7 (42). The VP1-98 residue that maps to positively charged patches around the 5-fold axis is also associated with Fab binding (42). Positive selection drives viruses to evolve in their favor, and residues VP1-98 and VP1-145 have been shown to produce mutants suitable for viral survival (43, 44). In addition, two data sets containing many sequences were found to have positive selection for amino acid residues VP1-184 (FG loop) and VP1-249 (HI loop). However, it has not been confirmed whether this correlates with virulence.

Although our study was limited by the lack of complete EV-A71 sequences for some years and regions, this work provides a basis for understanding the epidemiological characteristics, phylogenetic features, and Bayesian phylodynamics of EV-A71. While the EV-A71 genome is short, analysis of *VP1* alone cannot explain the epidemic characteristics of EV-A71. *3D^pol^*, a high-frequency recombination region of EV-A71, contains much epidemiological information on EV-A71 and needs to be studied. EV-A71 is widely prevalent in the Asia-Pacific region, and we performed a phylogeographic analysis in only some countries due to the limited number of sequences. B5 and C4 were introduced into other countries via Vietnam in this region, although they originated from different countries. Controlling the spread of the disease while promoting tourism and other industries is an issue of concern. The transmission routes of the widely prevalent C4 in China were mapped, with East China and North China playing a decisive role in spreading C4, guiding surveillance in key regions. In this study, two positive selection sites were identified, amino acid residues VP1-184 (FG loop) and VP1-249 (HI loop), which must be experimentally verified to determine whether they are associated with viral pathogenicity.

## MATERIALS AND METHODS

### Sample collection, ethical considerations, and virus isolation.

The national HFMD surveillance system was established in mainland China in 2008. All provinces, autonomous regions, and municipalities regularly report HFMD cases through the surveillance network, while representative clinical samples and sample information (case type, age, sex, and time of onset) are sent to the National Polio Laboratory for review via a standardized transport route. This study was conducted for public health surveillance, did not involve any human-related experiments, and was approved by the Second Ethics Review Committee of the National Institute for Viral Disease Control and Prevention (IVDC), Chinese Center for Disease Control and Prevention. Samples were collected from HFMD patients between 2016 and 2020, including stool samples, throat swabs, and anal swabs. Parents provided written informed consent. In addition, they were informed in detail about the entire sampling process and purpose of the specimens collected for the study. All clinical samples were handled in strict accordance with the WHO polio laboratory manual (4th edition, 45) and inoculated into human rhabdomyosarcoma (RD) and human laryngeal epidermoid carcinoma (HEp-2) cells (46). After complete EV-like cytopathic effects were observed, cell culture was collected.

### Molecular typing and whole-genome sequencing.

Viral RNA was extracted from cell cultures using the QIAamp Viral RNA minikit (Qiagen, Hilden, Germany), and positive samples were determined by real-time PCR (47). The positive specimens were amplified by reverse transcription-PCR (RT-PCR) using primers E486/E488, E490/E492, and E494/E496 with the PrimeScript one-step RT-PCR kit version 2 (TaKaRa, Dalian, China) to obtain partial *VP1* sequences (48). The products were purified using a QIAquick PCR purification kit (Qiagen, Hilden, Germany) and sequenced using an ABI 3130 genetic analyzer (Applied Biosystems, Foster City, CA, USA). The sequencing results were spliced to obtain partial *VP1* sequences using Sequencher software (v5.0). Enterovirus serotypes isolated from the specimens were determined by comparative analysis using the EV genotyping tool and BLAST server (49).

EV-A71 *VP1* sequences were selected and aligned using Muscle software (v3.8.31_i86linux32) (50), and the maximum-likelihood tree was constructed using RAxML software (v8.2.12) (51). Two hundred representative strains were selected from each branch of the evolutionary tree by combining sample collection time and geographical information for whole-genome sequencing (Fig. S1; Table S1). EV-A71 RNA was amplified by a 5′-Full RACE kit (TaKaRa, Shiga, Japan) to obtain the 5′ end, the 3′ end was obtained using an oligo(dT) primer (primer 7500A), and the remaining genome was obtained by designing specific primers using the primer-walking method (Table S2). Purification was performed using the QIAquick PCR purification kit (Qiagen, Hilden, Germany), and the genome was sequenced using the ABI 3130 genetic analyzer (Applied Biosystems, Foster City, CA, USA). The results were spliced using Sequencher software (v5.0).

### EV-A71 genome-wide data set construction.

The complete EV-A71 sequences from the GenBank database (length limited to 6,000 to 7,600 nt, as of 20 May 2020) excluded cloned sequences, multiple-passage sequences, and sequences with unknown dates or regions. Subsequently, the remaining sequences were aligned using Muscle software (v3.8.31_i86linux32). We removed sequences with many bases missing from the ORF, sequences containing a large number of N bases, and sequences that were not EV-A71. A data set containing 772 whole genomes of EV-A71 was obtained and combined with 200 newly sequenced EV-A71 from this study to form an EV-A71 data set containing 972 sequences.

A maximum-likelihood tree based on the 972 ORF sequence was constructed using RAxML software (v8.2.12), and the representative sequences of each branch were selected for subsequent study according to the virus isolation time combined with geographical distribution characteristics (Fig. S2). Finally, 259 EV-A71 sequences were selected for phylogenetic and recombination studies, and 85 B5 Asian, 170 C4 Asian, and 234 C4 China sequences were selected for phylogeographic analysis (Tables S3 to S7). The sequences were named according to the following principles: for Chinese isolates, isolate or GenBank number/two-letter provincial abbreviation/CHN/year of collection/subgenotype or lineage (e.g., D28/YN/CHN/2016/C4 for the sequence named D28 isolated from Yunnan, China, in 2016, which belongs to subgenotype C4); for isolates from other countries, GenBank number/three-letter country code/year of collection/subgenotype or lineage (e.g., DQ341361/AUS/2000/C1 represents the sequence isolated in Australia in 2000 with GenBank number DQ341361, which belongs to C1).

### Phylogenetic and recombination analysis.

The sequences of the data sets were aligned, and the best nucleotide substitution model was calculated for each data set using ModelGenerator 0.85 (52). The maximum-likelihood tree was constructed using the RAxML software (v8.2.12). Genetic diversity analysis was performed using the neighbor-joining method in MEGA (v11.0.11) (53). The Kimura 2-parameter model was chosen to calculate the intra- and intergroup mean distances using the bootstrap method, and the support was estimated with 1,000 bootstrap replicates. Recombination characteristics were analyzed for subgenotypes and lineages using the SimPlot software (v3.5.1) (54). Finally, sequences were imported into BioEdit (v7.0.9.0) to obtain the identity matrix (55), and the results are presented as a heat map using TBtool (v1.098726) (56).

### Temporal dynamics analysis.

Potential recombinants were detected using the RDP4 (v4.46) and SimPlot (v3.5.1) programs for 256 *VP1* sequences. RDP4 uses RDP, GENECONV, Chimaera, MaxChi, Bootscan, SiScan, and 3Seq, and significant recombination events were considered to exist when at least four methods were used (57). To examine the temporal structure of the data set, we performed a regression of root-to-tip genetic differences against the year of sampling in TempEst (v1.5) (58). In addition, we performed a data randomization test, generating 20 permutations of the sampling dates using the TipDating Beast package (59). Finally, the date-randomized replicates of the data set were analyzed using Bayesian phylogenetic methods in BEAST (v1.8.4) (60). The test results for each data set are presented in Fig. S3.

After validating the data set, we performed a Bayesian phylogenetic analysis using BEAST (v1.8.4). ModelGenerator (v0.85) calculated the optimal nucleotide substitution model for the data set, and the optimal model for the 256 sequences was SYM+I+G. Path sampling and stepping-stone sampling were used to analyze the best combination of the molecular clock model and tree prior (61), and 15 independent combinations (three different molecular clock models and five different tree priors) were used for each data set for comparison (Tables S8 to S11).

Trace software (v1.7.1) was used to verify the convergence of the parameters. The parameters were considered convergent when the effective sample size was >200 (62). The Bayesian MCC tree was constructed using TreeAnnotator software (v1.8.4), and the top 10% of the sampled trees were discarded using the burn-in option. Finally, the results were presented using FigTree software (v1.4).

### Geographical clustering intensity analysis by VP1 sequences.

We assigned the *VP1* region of each sequence of B5 and C4 a character state based on its sampling location, that is, different states in China or others. The B5 sampling area included Taiwan (China), Thailand, and Vietnam. C4 was analyzed in two parts; one included China, Cambodia, and Vietnam. The other was a division of China into eight regions: North China (Inner Mongolia, Shanxi, Hebei, Beijing, and Tianjin), Northeast China (Liaoning, Heilongjiang, and Jilin), Northwest China (Xinjiang, Qinghai, Gansu, Ningxia, and Shaanxi), Southwest China (Sichuan, Guizhou, Yunnan, Tibet, and Chongqing), East China (Shandong, Anhui, Jiangxi, Jiangsu, Zhejiang, Shanghai, and Fujian), South China (Guangxi, Guangdong, Hainan, Macau, and Hong Kong), Central China (Henan, Hubei, and Hunan), and Taiwan, China. BEAST software (v1.8.4) was used to perform Bayesian analysis and generate sample trees. BaTS (v2.0) was used to calculate three statistical parameters for the sample trees (63). The association index (AI) and parsimony score (PS) were used to determine the overall statistical significance of the geographical clustering of taxa in EV-A71 phylogenies. The maximum monophyletic clade (MC) compares the cluster strength of each group by calculating the expected and observed mean clade sizes for each group. A significance level of a *P* value of <0.05 was used as a judgment criterion for all three parameters to assess the strength of geographic clustering in EV-A71 data.

### Phylogeographic analysis by VP1 sequences.

To understand the prevalence of B5 and C4, we reconstructed EV-A71 spatial transmission patterns using phylogeographic analysis in BEAST software (v1.8.4). Based on the regional division of B5 and C4, regions were coded as discrete states, the substitution model was chosen as the asymmetric substitution model, and model averaging was performed using Bayesian stochastic search variable selection (64). The migration pathway, posterior probability (PP), and Bayes factor (BF) were calculated using SpreaD3 (v0.9.7.1) (65), and the migration pathway was considered significant when BF was >3 and PP was >0.50 (66, 67). Markov jump counts estimate the number of expected region-state transitions.

### Estimation of the *dN*/*dS* ratio in VP1 and 3D^pol^.

*dN*/*dS* is a valid indicator of the strength and mode of natural selection acting on protein-coding genes. The *dN*/*dS* ratio of different data sets was examined using single-likelihood ancestor counting (SLAC) and the mixed-effects model of evolution (MEME) on the Datamonkey website (68, 69) (http://www.Datamonkey.org).

The selection pressure was measured based on the *dN*/*dS* ratio for each *VP1*/*3D^pol^* codon, and *P* values were also calculated for these residues. The *P* value threshold was set to 0.1 to distinguish between positive and negative selection sites.

### Data availability.

Two hundred newly sequenced EV-A71 strains were uploaded to GenBank (accession numbers ON502178 to ON502377).
