# Supplementary material for: Genomic Epidemiology and Phylodynamic Analysis of Enterovirus A71 Reveal Its Transmission Dynamics in Asia
Source: Microbiol Spectr. 2022 Oct 6;10(5):e01958-22. doi: 10.1128/spectrum.01958-22 (PMC9603238; doi:10.1128/spectrum.01958-22)
Supplement: Supplemental file 1 — Supplemental material. Download spectrum.01958-22-s0001.pdf, PDF file, 1.3 MB [file spectrum.01958-22-s0001.pdf]

Genomic Epidemiology and Phylodynamic Analysis of EV-A71 Reveal Its Transmission Dynamics in Asia

Jinbo Xiao[1], Keqiang Huang[1], Huanhuan Lu[1], Yang Song[1], Zhenzhi Han[1], Man Zhang[2], Jichen Li[1], Xiaofang Zhou[3], Jianhua Chen[4], Qiuli Yu[5], Ming Yang[6], Dongmei Yan[1], Tianjiao Ji[1], Qian Yang[1], Shuangli Zhu[1], Wenbo Xu[1,7], Yong Zhang[1,7*]

[1]WHO WPRO Regional Polio Reference Laboratory and National Health Commission Key Laboratory of biosafety, National Institute for Viral Disease Control and Prevention, Chinese Center for Disease Control and Prevention, Beijing, People's Republic of China

[2]Shandong Center for Disease Control and Prevention, Jinan, Shandong Province, People's Republic of China

[3]Yunnan Center for Disease Control and Prevention, Kunming, Yunnan Province, People's Republic of China

[4]Gansu Center for Disease Control and Prevention, Lanzhou, Gansu Province, People's Republic of China

[5]Hebei Center for Disease Control and Prevention, Shijiazhuang, Hebei Province, People's Republic of China,

[6]Heilongjiang Center for Disease Control and Prevention, Harbin, Heilongjiang Province, People's Republic of China,

[7]Center for Biosafety Mega-Science, Chinese Academy of Sciences, Wuhan, Hubei Province, People's Republic of China

Supplementary Table S1. Geographic and temporal information of the 200 newly sequenced sequences.

Regions	Provinces	Numbers of sequences				Total	
		2016	2017	2018	2019		
North China	Shanxi		4	3		7	41
	Hebei	4	9	4	1	18	
	Beijing		2	5		7	
	Tianjin	5	1	3		9	
Northeast China	Liaoning		3			3	19
	Heilongjiang	4	2	5		11	
	Jilin			5		5	
Northwest China	Xinjiang	4		2		6	37
	Qinghai		2			2	
	Gansu	3	6	7	3	19	
	Ningxia		3	5		8	
	Shaanxi		2			2	
Southwest China	Sichuan	4				4	53
	Yunnan	32	5	3	3	43	
	Chongqing	4	2			6	
East China	Shandong	5	2		1	8	25
	Anhui	1	3			4	
	Jiangxi	5	2	1		8	
	Zhejiang	3	2			5	
South China	Guangxi		1			1	14
	Guangdong		2	4		6	
	Hainan		3	4		7	
Central China	Henan		3	3		6	11
	Hunan	5				5	
Total		79	59	54	8	200	

Supplementary Table S2. Primers used in this study for full-sequence amplification of EV-A71.

Primer name	Nucleotide position	Sequence (5'-3')	Orientation	Reference
0001S48		GGGGACAAGTTTGTACAAAAAAGCAGGCTTTAAAACAGCTCTGGGGTT	Forward	(1)
EV-A71-659A	659-678	CAATTGCTCTGTTGCACACC	Reverse	This study
EV-A71-1018A	1018-1037	GTGATGGTGGAGTTGCCAAT	Reverse	This study
EV-A71-863S	863-882	GGGCAAACAGAGTCTCAAGC	Forward	This study
EV-A71-1948A	1948-1967	GCGCAGTCTCTCCATTAAGC	Reverse	This study
EV-A71-1767S	1767-1786	GTTTCGGCACCTATTCTACC	Forward	This study
EV-A71-2578A	2578-2597	TGGAGTGCTGGAACCTTACC	Reverse	This study
EV-A71-2472S	2472-2491	ATAGGAGACAGCGTGAGTAG	Forward	This study
EV-A71-3423A	3423-3442	CGAGAACTGTCTTCCCAAAC	Reverse	This study
EV-A71-3342S	3342-3361	CAGTCTGGGGCCATTTATGT	Forward	This study
EV-A71-4345A	4345-4364	TGGAACTTGCGACAGAAGTG	Reverse	This study
EV-A71-4181S	4181-4200	AGTACCAGCAGCCAAGGAGA	Forward	This study
EV-A71-5207A	5207-5226	GGTGGGAGTTTCAGGAATGA	Reverse	This study
EV-A71-5068S	5068-5087	CACAATCGAGGCTCTTTTCC	Forward	This study
EV-A71-6195A	6195-6215	GCTGCCTCTTTGATGTACTCG	Reverse	This study
EV-A71-5897S	5897-5916	CGCAGGCCTTAAAAGGAGTT	Forward	This study
EV-A71-6749A	6749-6768	TGTGTGGTTGATTCCCTCAA	Reverse	This study
EV-A71-6602S	6602-6621	TTTGCTCCCTGGTTCACTCT	Forward	This study
7500A		GGGGACCACTTTGTACAAGAAAGCTGGG$(T)_{24}$	Reverse	(1)

Supplementary Table S3. Information on 259 global EV-A71 for phylogenetic analysis.

	Isolated year	Country	Source	Genotype/subgenotype	Evolutionary lineage
ZJ/10	2016	China	This study	C4	I
ZJ/110	2017	China	This study	C4	I
BJ/13	2018	China	This study	C4	I
XJ/13	2018	China	This study	C4	I
TJ/1321Y	2016	China	This study	C4	I
SX/164	2017	China	This study	C4	I
GS/165	2018	China	This study	C4	I
BJ/17	2017	China	This study	C4	I
HB/181	2017	China	This study	C4	I
BJ/19	2018	China	This study	C4	I
HaN/19	2017	China	This study	C4	I
HLJ/19	2017	China	This study	C4	I
BJ/22	2017	China	This study	C4	I
GD/22	2018	China	This study	C4	I
SD/238	2017	China	This study	C4	I
HaN/24	2018	China	This study	C4	I
HeN/250	2018	China	This study	C4	I
JX/26	2018	China	This study	C4	I
LN/26-67	2017	China	This study	C4	I
HB/269	2018	China	This study	C4	I
HeN/276	2017	China	This study	C4	I
HeN/287	2017	China	This study	C4	I
XJ/3	2018	China	This study	C4	I
HeN/313	2017	China	This study	C4	I
GS/373	2017	China	This study	C4	I
HuN/39	2016	China	This study	C4	I
JX/39	2017	China	This study	C4	I
CQ/4	2017	China	This study	C4	I
AH/50	2017	China	This study	C4	I
SC/50	2016	China	This study	C4	I
GS/520	2017	China	This study	C4	I
TJ/53	2017	China	This study	C4	I
GD/58	2018	China	This study	C4	I
JX/59	2016	China	This study	C4	I
HaN/6	2017	China	This study	C4	I
QH/63	2017	China	This study	C4	I
AH/65	2017	China	This study	C4	I
HLJ/66	2018	China	This study	C4	I
CQ/68	2016	China	This study	C4	I
SX/69	2018	China	This study	C4	I
HLJ/71	2018	China	This study	C4	I
GD/8	2017	China	This study	C4	I

JL/83	2018	China	This study	C4	I
XJ/83	2016	China	This study	C4	I
SX/86	2017	China	This study	C4	I
BJ/9	2018	China	This study	C4	I
GS/9	2016	China	This study	C4	I
XJ/93	2016	China	This study	C4	I
HeN/a5	2018	China	This study	C4	I
YN/A50	2017	China	This study	C4	I
YN/A80	2018	China	This study	C4	I
AB575911	1966	Nederlands	Genbank	B0	B
AB575912	1966	Nederlands	Genbank	B0	B
AB575935	1991	Nederlands	Genbank	C1	E
AB575937	2001	Nederlands	Genbank	C1	E
AB575942	2007	Nederlands	Genbank	C2	E
AF136379	1998	China	Genbank	C2	E
AF304457	1998	China	Genbank	C2	E
AM396585	1997	Malaysia	Genbank	C2	E
AM396586	1997	Malaysia	Genbank	B3	F
YN/B89	2017	China	This study	C4	I
YN/C76	2018	China	This study	C4	I
YN/C99	2018	China	This study	C4	I
YN/D28	2016	China	This study	C4	I
DQ341354	1998	Singapore	Genbank	B3	F
DQ341355	2000	Korea	Genbank	C3	G
DQ341356	2000	Korea	Genbank	C3	G
DQ341357	1999	Australia	Genbank	C2	E
DQ341358	2000	Malaysia	Genbank	C1	E
DQ341359	1998	Malaysia	Genbank	C1	E
DQ341361	2000	Australia	Genbank	C1	E
DQ341363	2003	Malaysia	Genbank	B5	H
DQ341365	2001	Malaysia	Genbank	B4	H
DQ341368	1997	Malaysia	Genbank	B3	F
DQ381846	1999	Australia	Genbank	C2	E
EF063152	2006	China	Genbank	C5	L
TJ/ET0520	2016	China	Genbank	C4	I
EU364841	1999	Australia	Genbank	B3	F
EU527983	2007	China	Genbank	C5	L
EU703812	2008	China	Genbank	C4	I
EU703813	2008	China	Genbank	C4	I
YN/F257	2016	China	This study	C4	I
HuN/F55	2016	China	This study	C4	I
HuN/FC47	2016	China	This study	C4	I
FJ172159	2008	Singapore	Genbank	C2	E
FJ194965	2008	China	Genbank	C4	I

FJ357373	2004	China	Genbank	C4	I
FJ357376	2001	China	Genbank	B4	H
FJ357378	2003	China	Genbank	B5	H
FJ357380	1986	China	Genbank	B1	C
FJ357381	1986	China	Genbank	B1	C
FJ360545	2008	China	Genbank	C4	I
FJ461781	2008	Singapore	Genbank	B5	H
FJ606450	2008	China	Genbank	C4	N
FJ713137	2009	China	Genbank	C4	I
GQ231929	2004	China	Genbank	C4	I
GQ231935	2008	China	Genbank	B5	H
GQ231936	2008	China	Genbank	B5	H
GU459071	2008	China	Genbank	C4	I
NX/GY038	2017	China	This study	C4	I
NY/GY096	2017	China	This study	C4	I
HM156065	2009	China	Genbank	B5	H
HM245927	2010	China	Genbank	C4	I
HM622390	2009	China	Genbank	B5	H
HM622391	2008	China	Genbank	C2	M
HM622392	2008	China	Genbank	C2	M
HQ426649	2010	China	Genbank	C4	I
HQ647167	2007	Canada	Genbank	C2	E
HQ647177	2007	Canada	Genbank	C2	E
HQ647179	2006	Canada	Genbank	C4	I
HQ694984	2008	China	Genbank	C4	I
HQ694985	2008	China	Genbank	C4	I
HQ825317	2008	China	Genbank	C4	I
HQ891923	2009	China	Genbank	C4	I
YN/I12	2019	China	This study	C4	I
YN/I13	2016	China	This study	C4	I
YN/J48	2017	China	This study	C4	I
JF738000	2009	Thailand	Genbank	C1	E
JF820312	2010	China	Genbank	C4	I
JF830007	2010	China	Genbank	C4	I
JN052925	2011	China	Genbank	C4	I
JN864019	2010	China	Genbank	C4	I
JQ280307	2008	China	Genbank	C2	M
JQ319054	2009	China	Genbank	C4	I
JQ639384	2011	China	Genbank	C4	I
JQ681218	2008	China	Genbank	C4	I
JQ742002	2001	China	Genbank	C4	I
JX244182	2009	China	Genbank	C4	I
JX244184	2009	China	Genbank	C4	I
JX244187	2010	China	Genbank	C4	I

HLJ/JX80	2016	China	This study	C4	I
KC296443	2011	Vietnam	Genbank	C4	I
KC296444	2011	Vietnam	Genbank	C5	L
KC296445	2011	Vietnam	Genbank	C4	I
KC414134	2011	China	Genbank	C4	O
KF142412	2013	China	Genbank	C4	I
KF142413	2013	China	Genbank	C4	I
KF543271	2012	Cambodia	Genbank	C4	I
KF974782	2008	China	Genbank	B5	H
KF974793	2012	China	Genbank	B5	H
KF974794	2012	China	Genbank	B5	H
KF974796	2012	China	Genbank	B5	H
KJ186973	2010	China	Genbank	C4	I
KJ400360	2013	Russia	Genbank	C4	I
KJ686131	2012	Vietnam	Genbank	B5	H
KJ686137	2012	Vietnam	Genbank	B5	H
KJ686144	2012	Vietnam	Genbank	C5	L
KJ686155	2012	Vietnam	Genbank	C4	I
KJ686165	2011	Vietnam	Genbank	C4	I
KJ686176	2012	Vietnam	Genbank	B5	H
KJ686178	2011	Vietnam	Genbank	C5	L
KJ686179	2011	Vietnam	Genbank	C5	L
KJ686190	2011	Vietnam	Genbank	C4	I
KJ686200	2011	Vietnam	Genbank	C4	I
KJ686220	2012	Vietnam	Genbank	C4	I
KJ686230	2011	Vietnam	Genbank	C5	L
KJ686239	2011	Vietnam	Genbank	C4	I
KJ686240	2012	Vietnam	Genbank	C4	I
KJ686252	2012	Vietnam	Genbank	C4	I
KJ686254	2012	Vietnam	Genbank	C4	I
KJ686255	2011	Vietnam	Genbank	C4	I
KJ686259	2012	Vietnam	Genbank	C4	I
KJ686274	2012	Vietnam	Genbank	C4	I
KJ686285	2012	Vietnam	Genbank	C4	I
KJ686290	2011	Vietnam	Genbank	C4	I
KJ686293	2012	Vietnam	Genbank	C4	I
KJ686303	2011	Vietnam	Genbank	C4	I
KJ686306	2012	Vietnam	Genbank	C4	I
KJ784495	2011	China	Genbank	C4	O
KJ784496	2012	China	Genbank	C4	I
KM055005	2011	Laos	Genbank	C4	I
KP198624	2012	China	Genbank	C4	I
KP274875	2011	China	Genbank	C4	I
KP274877	2011	China	Genbank	B4	H

KP289418	2013	China	Genbank	C4	I
KP308403	2012	Cambodia	Genbank	C4	I
KP308409	2012	Cambodia	Genbank	C4	I
KP308423	2012	Cambodia	Genbank	C4	I
KP308428	2012	Cambodia	Genbank	C4	I
KP308430	2012	Cambodia	Genbank	B5	H
KP308438	2012	Cambodia	Genbank	C4	I
KP308449	2012	Cambodia	Genbank	C4	I
KP308451	2012	Cambodia	Genbank	C4	I
KP308459	2012	Cambodia	Genbank	C4	I
KP691658	2013	Vietnam	Genbank	B5	H
KP691665	2011	Vietnam	Genbank	C4	I
KR045297	2012	Thailand	Genbank	B5	H
KR045298	2012	Thailand	Genbank	B5	H
KR045301	2014	Thailand	Genbank	B5	H
KU254596	2014	China	Genbank	C4	I
KU641507	2015	Germany	Genbank	C1	Q
KU641508	2015	Germany	Genbank	C1	Q
KX139462	2015	Germany	Genbank	C1	Q
KX197460	2012	Cambodia	Genbank	C4	I
KX197464	2012	Cambodia	Genbank	C4	I
KX197465	2012	Cambodia	Genbank	C4	I
KX372308	2006	Thailand	Genbank	C4	K
KX372309	2008	Thailand	Genbank	C4	K
KX372316	2012	Thailand	Genbank	B5	H
KX372319	2012	Thailand	Genbank	B5	H
KX372324	2013	Thailand	Genbank	C2	E
KX430823	2014	Vietnam	Genbank	B5	H
KY014080	2008	China	Genbank	C4	I
KY425527	2013	China	Genbank	C4	I
KY888025	2016	United States	Genbank	C2	E
KY888026	2016	United States	Genbank	C1	Q
LC321990	2017	Japan	Genbank	C1	Q
LT719065	2004	Madagascaragascar	Genbank	F	J
LT719066	2008	Cameroon	Genbank	F	J
LT719067	2008	Cameroon	Genbank	C1	E
MF362981	2010	China	Genbank	C4	I
MG367596	2016	Danmark	Genbank	C1	Q
MG367598	2016	Danmark	Genbank	C1	Q
MG432108	1987	Singapore	Genbank	B2	D
MG672478	2013	Nigeria	Genbank	E	P
MG756691	2016	China	Genbank	C4	I
MG756695	2015	China	Genbank	B5	H
MG756699	2005	China	Genbank	C4	I

MG756703	2005	China	Genbank	C4	I
MG756706	2008	China	Genbank	B5	H
MG756720	2011	China	Genbank	C4	I
MG756722	2011	China	Genbank	C4	I
MG756746	2012	China	Genbank	B5	H
MG773122	2014	China	Genbank	C4	I
MG773123	2014	China	Genbank	C4	I
MG976581	2016	United States	Genbank	C2	E
MH718269	2018	United States	Genbank	C1	Q
YN/P35	2019	China	This study	C4	I
YN/P53	2019	China	This study	C4	I
SD/RZ151	2016	China	This study	C4	I
SD/RZ313	2016	China	This study	C4	I
GS/SC158	2019	China	This study	C4	I
HB/SC232	2017	China	This study	C4	I
GS/SC291	2019	China	This study	C4	I
HB/SC358	2017	China	This study	C4	I
HB/SC375	2017	China	This study	C4	I
HB/SC376	2017	China	This study	C4	I
HB/SC39	2019	China	This study	C4	I
GS/SC582	2018	China	This study	C4	I
YN/SCF256	2016	China	This study	C4	I
YN/SCF259	2016	China	This study	C4	I
YN/SCF264	2016	China	This study	C4	I
YN/SCF276	2016	China	This study	C4	I
YN/SCF284	2016	China	This study	C4	I
YN/SCF285	2016	China	This study	C4	I
YN/SCF288	2016	China	This study	C4	I
YN/SCF292	2016	China	This study	C4	I
YN/SCF303	2016	China	This study	C4	I
YN/SCF314	2016	China	This study	C4	I
YN/SCF327	2016	China	This study	C4	I
YN/SCF329	2016	China	This study	C4	I
NX/SZS47	2018	China	This study	C4	I
U22521/BrCr	1970	United States	Genbank	A	A
U22522	1987	United States	Genbank	B2	D
SD/WF305	2019	China	This study	C4	I
NX/WZ94	2018	China	This study	C4	I
CQ/Y86	2016	China	This study	C4	I
NX/YC188	2018	China	This study	C4	I
SD/YT194	2016	China	This study	C4	I

Supplementary Table S4. Information on 256 global EV-A71 for temporal dynamics analysis.

Strain name/Genbank No.	Isolated year	Country	Source	Genotype/subgenotype	Evolutionary lineage
ZJ/10	2016	China	This study	C4	I
ZJ/110	2017	China	This study	C4	I
BJ/13	2018	China	This study	C4	I
XJ/13	2018	China	This study	C4	I
TJ/1321Y	2016	China	This study	C4	I
SX/164	2017	China	This study	C4	I
GS/165	2018	China	This study	C4	I
BJ/17	2017	China	This study	C4	I
HB/181	2017	China	This study	C4	I
BJ/19	2018	China	This study	C4	I
HaN/19	2017	China	This study	C4	I
HLJ/19	2017	China	This study	C4	I
BJ/22	2017	China	This study	C4	I
GD/22	2018	China	This study	C4	I
SD/238	2017	China	This study	C4	I
HaN/24	2018	China	This study	C4	I
HeN/250	2018	China	This study	C4	I
JX/26	2018	China	This study	C4	I
LN/26-67	2017	China	This study	C4	I
HB/269	2018	China	This study	C4	I
HeN/276	2017	China	This study	C4	I
HeN/287	2017	China	This study	C4	I
XJ/3	2018	China	This study	C4	I
HeN/313	2017	China	This study	C4	I
GS/373	2017	China	This study	C4	I
HuN/39	2016	China	This study	C4	I
JX/39	2017	China	This study	C4	I
CQ/4	2017	China	This study	C4	I
AH/50	2017	China	This study	C4	I
SC/50	2016	China	This study	C4	I
GS/520	2017	China	This study	C4	I
TJ/53	2017	China	This study	C4	I
GD/58	2018	China	This study	C4	I
JX/59	2016	China	This study	C4	I
HaN/6	2017	China	This study	C4	I
QH/63	2017	China	This study	C4	I
AH/65	2017	China	This study	C4	I
HLJ/66	2018	China	This study	C4	I
CQ/68	2016	China	This study	C4	I
SX/69	2018	China	This study	C4	I
HLJ/71	2018	China	This study	C4	I

GD/8	2017	China	This study	C4	I
JL/83	2018	China	This study	C4	I
XJ/83	2016	China	This study	C4	I
SX/86	2017	China	This study	C4	I
BJ/9	2018	China	This study	C4	I
GS/9	2016	China	This study	C4	I
XJ/93	2016	China	This study	C4	I
HeN/a5	2018	China	This study	C4	I
YN/A50	2017	China	This study	C4	I
YN/A80	2018	China	This study	C4	I
AB575911	1966	Nederlands	Genbank	B0	B
AB575912	1966	Nederlands	Genbank	B0	B
AB575935	1991	Nederlands	Genbank	C1	E
AB575937	2001	Nederlands	Genbank	C1	E
AB575942	2007	Nederlands	Genbank	C2	E
AF136379	1998	China	Genbank	C2	E
AF304457	1998	China	Genbank	C2	E
AM396585	1997	Malaysia	Genbank	C2	E
AM396586	1997	Malaysia	Genbank	B3	F
YN/B89	2017	China	This study	C4	I
YN/C76	2018	China	This study	C4	I
YN/C99	2018	China	This study	C4	I
YN/D28	2016	China	This study	C4	I
DQ341354	1998	Singapore	Genbank	B3	F
DQ341355	2000	Korea	Genbank	C3	G
DQ341356	2000	Korea	Genbank	C3	G
DQ341357	1999	Australia	Genbank	C2	E
DQ341358	2000	Malaysia	Genbank	C1	E
DQ341359	1998	Malaysia	Genbank	C1	E
DQ341361	2000	Australia	Genbank	C1	E
DQ341363	2003	Malaysia	Genbank	B5	H
DQ341365	2001	Malaysia	Genbank	B4	H
DQ341368	1997	Malaysia	Genbank	B3	F
DQ381846	1999	Australia	Genbank	C2	E
EF063152	2006	China	Genbank	C5	L
TJ/ET0520	2016	China	Genbank	C4	I
EU364841	1999	Australia	Genbank	B3	F
EU527983	2007	China	Genbank	C5	L
EU703812	2008	China	Genbank	C4	I
EU703813	2008	China	Genbank	C4	I
YN/F257	2016	China	This study	C4	I
HuN/F55	2016	China	This study	C4	I
HuN/FC47	2016	China	This study	C4	I
FJ172159	2008	Singapore	Genbank	C2	E

FJ194965	2008	China	Genbank	C4	I
FJ357373	2004	China	Genbank	C4	I
FJ357376	2001	China	Genbank	B4	H
FJ357378	2003	China	Genbank	B5	H
FJ357380	1986	China	Genbank	B1	C
FJ357381	1986	China	Genbank	B1	C
FJ360545	2008	China	Genbank	C4	I
FJ461781	2008	Singapore	Genbank	B5	H
FJ606450	2008	China	Genbank	C4	N
FJ713137	2009	China	Genbank	C4	I
GQ231929	2004	China	Genbank	C4	I
GQ231935	2008	China	Genbank	B5	H
GQ231936	2008	China	Genbank	B5	H
GU459071	2008	China	Genbank	C4	I
NX/GY038	2017	China	This study	C4	I
NY/GY096	2017	China	This study	C4	I
HM156065	2009	China	Genbank	B5	H
HM245927	2010	China	Genbank	C4	I
HM622390	2009	China	Genbank	B5	H
HM622391	2008	China	Genbank	C2	M
HM622392	2008	China	Genbank	C2	M
HQ426649	2010	China	Genbank	C4	I
HQ647167	2007	Canada	Genbank	C2	E
HQ647177	2007	Canada	Genbank	C2	E
HQ647179	2006	Canada	Genbank	C4	I
HQ694984	2008	China	Genbank	C4	I
HQ694985	2008	China	Genbank	C4	I
HQ825317	2008	China	Genbank	C4	I
HQ891923	2009	China	Genbank	C4	I
YN/I12	2019	China	This study	C4	I
YN/I13	2016	China	This study	C4	I
YN/J48	2017	China	This study	C4	I
JF738000	2009	Thailand	Genbank	C1	E
JF820312	2010	China	Genbank	C4	I
JF830007	2010	China	Genbank	C4	I
JN052925	2011	China	Genbank	C4	I
JN864019	2010	China	Genbank	C4	I
JQ280307	2008	China	Genbank	C2	M
JQ319054	2009	China	Genbank	C4	I
JQ639384	2011	China	Genbank	C4	I
JQ681218	2008	China	Genbank	C4	I
JQ742002	2001	China	Genbank	C4	I
JX244182	2009	China	Genbank	C4	I
JX244184	2009	China	Genbank	C4	I

JX244187	2010	China	Genbank	C4	I
HLJ/JX80	2016	China	This study	C4	I
KC296443	2011	Vietnam	Genbank	C4	I
KC296444	2011	Vietnam	Genbank	C5	L
KC296445	2011	Vietnam	Genbank	C4	I
KC414134	2011	China	Genbank	C4	O
KF142412	2013	China	Genbank	C4	I
KF142413	2013	China	Genbank	C4	I
KF543271	2012	Cambodia	Genbank	C4	I
KF974782	2008	China	Genbank	B5	H
KF974793	2012	China	Genbank	B5	H
KF974794	2012	China	Genbank	B5	H
KF974796	2012	China	Genbank	B5	H
KJ186973	2010	China	Genbank	C4	I
KJ400360	2013	Russia	Genbank	C4	I
KJ686131	2012	Vietnam	Genbank	B5	H
KJ686137	2012	Vietnam	Genbank	B5	H
KJ686144	2012	Vietnam	Genbank	C5	L
KJ686155	2012	Vietnam	Genbank	C4	I
KJ686165	2011	Vietnam	Genbank	C4	I
KJ686176	2012	Vietnam	Genbank	B5	H
KJ686178	2011	Vietnam	Genbank	C5	L
KJ686179	2011	Vietnam	Genbank	C5	L
KJ686190	2011	Vietnam	Genbank	C4	I
KJ686200	2011	Vietnam	Genbank	C4	I
KJ686220	2012	Vietnam	Genbank	C4	I
KJ686230	2011	Vietnam	Genbank	C5	L
KJ686239	2011	Vietnam	Genbank	C4	I
KJ686240	2012	Vietnam	Genbank	C4	I
KJ686252	2012	Vietnam	Genbank	C4	I
KJ686254	2012	Vietnam	Genbank	C4	I
KJ686255	2011	Vietnam	Genbank	C4	I
KJ686259	2012	Vietnam	Genbank	C4	I
KJ686274	2012	Vietnam	Genbank	C4	I
KJ686285	2012	Vietnam	Genbank	C4	I
KJ686290	2011	Vietnam	Genbank	C4	I
KJ686293	2012	Vietnam	Genbank	C4	I
KJ686303	2011	Vietnam	Genbank	C4	I
KJ686306	2012	Vietnam	Genbank	C4	I
KJ784495	2011	China	Genbank	C4	O
KJ784496	2012	China	Genbank	C4	I
KM055005	2011	Laos	Genbank	C4	I
KP198624	2012	China	Genbank	C4	I
KP274875	2011	China	Genbank	C4	I

KP274877	2011	China	Genbank	B4	H
KP289418	2013	China	Genbank	C4	I
KP308403	2012	Cambodia	Genbank	C4	I
KP308409	2012	Cambodia	Genbank	C4	I
KP308423	2012	Cambodia	Genbank	C4	I
KP308428	2012	Cambodia	Genbank	C4	I
KP308430	2012	Cambodia	Genbank	B5	H
KP308438	2012	Cambodia	Genbank	C4	I
KP308449	2012	Cambodia	Genbank	C4	I
KP308451	2012	Cambodia	Genbank	C4	I
KP308459	2012	Cambodia	Genbank	C4	I
KP691658	2013	Vietnam	Genbank	B5	H
KP691665	2011	Vietnam	Genbank	C4	I
KR045297	2012	Thailand	Genbank	B5	H
KR045298	2012	Thailand	Genbank	B5	H
KR045301	2014	Thailand	Genbank	B5	H
KU254596	2014	China	Genbank	C4	I
KU641507	2015	Germany	Genbank	C1	Q
KU641508	2015	Germany	Genbank	C1	Q
KX139462	2015	Germany	Genbank	C1	Q
KX197460	2012	Cambodia	Genbank	C4	I
KX197464	2012	Cambodia	Genbank	C4	I
KX197465	2012	Cambodia	Genbank	C4	I
KX372308	2006	Thailand	Genbank	C4	K
KX372309	2008	Thailand	Genbank	C4	K
KX372316	2012	Thailand	Genbank	B5	H
KX372319	2012	Thailand	Genbank	B5	H
KX372324	2013	Thailand	Genbank	C2	E
KX430823	2014	Vietnam	Genbank	B5	H
KY014080	2008	China	Genbank	C4	I
KY425527	2013	China	Genbank	C4	I
KY888025	2016	United States	Genbank	C2	E
KY888026	2016	United States	Genbank	C1	Q
LC321990	2017	Japan	Genbank	C1	Q
LT719067	2008	Cameroon	Genbank	C1	E
MF362981	2010	China	Genbank	C4	I
MG367596	2016	Danmark	Genbank	C1	Q
MG367598	2016	Danmark	Genbank	C1	Q
MG432108	1987	Singapore	Genbank	B2	D
MG672478	2013	Nigeria	Genbank	E	P
MG756691	2016	China	Genbank	C4	I
MG756695	2015	China	Genbank	B5	H
MG756699	2005	China	Genbank	C4	I
MG756703	2005	China	Genbank	C4	I

MG756706	2008	China	Genbank	B5	H
MG756720	2011	China	Genbank	C4	I
MG756722	2011	China	Genbank	C4	I
MG756746	2012	China	Genbank	B5	H
MG773122	2014	China	Genbank	C4	I
MG773123	2014	China	Genbank	C4	I
MG976581	2016	United States	Genbank	C2	E
MH718269	2018	United States	Genbank	C1	Q
YN/P35	2019	China	This study	C4	I
YN/P53	2019	China	This study	C4	I
SD/RZ151	2016	China	This study	C4	I
SD/RZ313	2016	China	This study	C4	I
GS/SC158	2019	China	This study	C4	I
HB/SC232	2017	China	This study	C4	I
GS/SC291	2019	China	This study	C4	I
HB/SC358	2017	China	This study	C4	I
HB/SC375	2017	China	This study	C4	I
HB/SC376	2017	China	This study	C4	I
HB/SC39	2019	China	This study	C4	I
GS/SC582	2018	China	This study	C4	I
YN/SCF256	2016	China	This study	C4	I
YN/SCF259	2016	China	This study	C4	I
YN/SCF264	2016	China	This study	C4	I
YN/SCF276	2016	China	This study	C4	I
YN/SCF284	2016	China	This study	C4	I
YN/SCF285	2016	China	This study	C4	I
YN/SCF288	2016	China	This study	C4	I
YN/SCF292	2016	China	This study	C4	I
YN/SCF303	2016	China	This study	C4	I
YN/SCF314	2016	China	This study	C4	I
YN/SCF327	2016	China	This study	C4	I
YN/SCF329	2016	China	This study	C4	I
NX/SZS47	2018	China	This study	C4	I
U22522	1987	United States	Genbank	B2	D
SD/WF305	2019	China	This study	C4	I
NX/WZ94	2018	China	This study	C4	I
CQ/Y86	2016	China	This study	C4	I
NX/YC188	2018	China	This study	C4	I
SD/YT194	2016	China	This study	C4	I

Supplementary Table S5. Information on 85 Asian B5 for phylogeographic analysis.

Strain name/Genbank No.	Isolated year	Country	Source	Genotype/subgenotype	Evolutionary lineage
EU527985	2007	Taiwan of China	Genbank	B5	H
GQ231925	2008	Taiwan of China	Genbank	B5	H
GQ231935	2008	Taiwan of China	Genbank	B5	H
GQ231941	2008	Taiwan of China	Genbank	B5	H
HM156065	2009	Taiwan of China	Genbank	B5	H
HM622390	2009	Taiwan of China	Genbank	B5	H
JF738001	2009	Thailand	Genbank	B5	H
KF134486	2011	Taiwan of China	Genbank	B5	H
KF154355	2012	Taiwan of China	Genbank	B5	H
KF306100	2011	Taiwan of China	Genbank	B5	H
KF974779	2008	Taiwan of China	Genbank	B5	H
KF974781	2008	Taiwan of China	Genbank	B5	H
KF974786	2008	Taiwan of China	Genbank	B5	H
KF974789	2008	Taiwan of China	Genbank	B5	H
KF974791	2012	Taiwan of China	Genbank	B5	H
KF974794	2012	Taiwan of China	Genbank	B5	H
KF974797	2012	Taiwan of China	Genbank	B5	H
KJ686128	2012	Vietnam	Genbank	B5	H
KJ686131	2012	Vietnam	Genbank	B5	H
KJ686137	2012	Vietnam	Genbank	B5	H
KJ686140	2012	Vietnam	Genbank	B5	H
KJ686176	2012	Vietnam	Genbank	B5	H
KJ686192	2012	Vietnam	Genbank	B5	H
KJ686211	2012	Vietnam	Genbank	B5	H
KJ686222	2012	Vietnam	Genbank	B5	H
KJ686225	2012	Vietnam	Genbank	B5	H
KJ686234	2012	Vietnam	Genbank	B5	H
KJ686264	2012	Vietnam	Genbank	B5	H
KJ686270	2012	Vietnam	Genbank	B5	H
KJ686277	2012	Vietnam	Genbank	B5	H
KJ686296	2012	Vietnam	Genbank	B5	H
KJ686297	2011	Vietnam	Genbank	B5	H
KJ686302	2012	Vietnam	Genbank	B5	H
KP691648	2013	Vietnam	Genbank	B5	H
KP691653	2013	Vietnam	Genbank	B5	H
KP691658	2013	Vietnam	Genbank	B5	H
KP691661	2013	Vietnam	Genbank	B5	H
KR045291	2012	Thailand	Genbank	B5	H
KR045292	2012	Thailand	Genbank	B5	H
KR045293	2012	Thailand	Genbank	B5	H
KR045294	2012	Thailand	Genbank	B5	H
KR045295	2012	Thailand	Genbank	B5	H

KR045296	2012	Thailand	Genbank	B5	H
KR045297	2012	Thailand	Genbank	B5	H
KR045298	2012	Thailand	Genbank	B5	H
KR045299	2012	Thailand	Genbank	B5	H
KR045300	2013	Thailand	Genbank	B5	H
KR045301	2014	Thailand	Genbank	B5	H
KU574619	2011	Thailand	Genbank	B5	H
KX372310	2011	Thailand	Genbank	B5	H
KX372311	2011	Thailand	Genbank	B5	H
KX372312	2011	Thailand	Genbank	B5	H
KX372313	2011	Thailand	Genbank	B5	H
KX372314	2012	Thailand	Genbank	B5	H
KX372315	2012	Thailand	Genbank	B5	H
KX372316	2012	Thailand	Genbank	B5	H
KX372317	2012	Thailand	Genbank	B5	H
KX372318	2012	Thailand	Genbank	B5	H
KX372319	2012	Thailand	Genbank	B5	H
KX372320	2012	Thailand	Genbank	B5	H
KX372321	2012	Thailand	Genbank	B5	H
KX372323	2013	Thailand	Genbank	B5	H
KX372325	2013	Thailand	Genbank	B5	H
KX372326	2013	Thailand	Genbank	B5	H
KX372327	2013	Thailand	Genbank	B5	H
KX372329	2014	Thailand	Genbank	B5	H
KX372330	2014	Thailand	Genbank	B5	H
KX372331	2014	Thailand	Genbank	B5	H
KX430823	2014	Vietnam	Genbank	B5	H
KX430824	2014	Vietnam	Genbank	B5	H
KY952186	2011	Thailand	Genbank	B5	H
MG756694	2015	Taiwan of China	Genbank	B5	H
MG756695	2015	Taiwan of China	Genbank	B5	H
MG756706	2008	Taiwan of China	Genbank	B5	H
MG756709	2008	Taiwan of China	Genbank	B5	H
MG756712	2008	Taiwan of China	Genbank	B5	H
MG756721	2011	Taiwan of China	Genbank	B5	H
MG756723	2011	Taiwan of China	Genbank	B5	H
MG756728	2012	Taiwan of China	Genbank	B5	H
MG756732	2012	Taiwan of China	Genbank	B5	H
MG756736	2012	Taiwan of China	Genbank	B5	H
MG756744	2012	Taiwan of China	Genbank	B5	H
MG756748	2012	Taiwan of China	Genbank	B5	H
MG756750	2012	Taiwan of China	Genbank	B5	H
MG756754	2013	Taiwan of China	Genbank	B5	H

Supplementary Table S6. Information on 170 Asian C4 for phylogeographic analysis.

Strain name/Genbank No.	Isolated year	Country	Source	Genotype/subgenotype	Evolutionary lineage
LN/11-11	2017	China	This study	C4	I
XJ/13	2018	China	This study	C4	I
SX/164	2017	China	This study	C4	I
SC/17	2016	China	This study	C4	I
AH/2	2016	China	This study	C4	I
JL/20	2018	China	This study	C4	I
JX/21	2017	China	This study	C4	I
GD/22	2018	China	This study	C4	I
HuN/39	2016	China	This study	C4	I
EU131776	2002	China	Genbank	C4	I
EU812515	2008	China	Genbank	C4	I
FJ606449	2008	China	Genbank	C4	I
FJ606450	2008	China	Genbank	C4	N
FJ607337	2008	China	Genbank	C4	N
GQ231926	2005	China	Genbank	C4	I
GQ231928	2004	China	Genbank	C4	I
GQ231930	2004	China	Genbank	C4	I
GQ994989	2009	China	Genbank	C4	I
GU198369	2008	China	Genbank	C4	I
GU396280	2009	China	Genbank	C4	I
HM245927	2010	China	Genbank	C4	I
HQ129932	2006	China	Genbank	C4	I
HQ423142	2009	China	Genbank	C4	O
HQ423143	2009	China	Genbank	C4	O
HQ694986	2010	China	Genbank	C4	I
YN/I12	2019	China	This study	C4	I
JF894381	2011	China	Genbank	C4	I
JQ742001	2001	China	Genbank	C4	I
JQ742002	2001	China	Genbank	C4	I
JX111888	2011	China	Genbank	C4	I
JX678874	2003	China	Genbank	C4	I
JX678882	2007	China	Genbank	C4	I
JX678885	2002	China	Genbank	C4	I
JX678886	2002	China	Genbank	C4	I
KC296443	2011	Vietnam	Genbank	C4	I
KC414134	2011	China	Genbank	C4	O
KC436265	2004	China	Genbank	C4	I
KC436267	2006	China	Genbank	C4	I
KC436268	2007	China	Genbank	C4	I
KC436271	2009	China	Genbank	C4	I
KC570452	2012	China	Genbank	C4	I
KC954662	2007	China	Genbank	C4	I

KF142411	2013	China	Genbank	C4	I
KF543271	2012	Cambodia	Genbank	C4	I
KF826491	2012	China	Genbank	C4	I
KJ004554	2012	China	Genbank	C4	I
KJ186974	2011	China	Genbank	C4	I
KJ508182	2012	China	Genbank	C4	I
KJ632498	2011	China	Genbank	C4	I
KJ686127	2011	Vietnam	Genbank	C4	I
KJ686129	2012	Vietnam	Genbank	C4	I
KJ686135	2012	Vietnam	Genbank	C4	I
KJ686136	2011	Vietnam	Genbank	C4	I
KJ686139	2012	Vietnam	Genbank	C4	I
KJ686143	2011	Vietnam	Genbank	C4	I
KJ686147	2011	Vietnam	Genbank	C4	I
KJ686148	2012	Vietnam	Genbank	C4	I
KJ686150	2012	Vietnam	Genbank	C4	I
KJ686154	2011	Vietnam	Genbank	C4	I
KJ686155	2012	Vietnam	Genbank	C4	I
KJ686160	2012	Vietnam	Genbank	C4	I
KJ686164	2011	Vietnam	Genbank	C4	I
KJ686166	2011	Vietnam	Genbank	C4	I
KJ686169	2011	Vietnam	Genbank	C4	I
KJ686173	2011	Vietnam	Genbank	C4	I
KJ686180	2012	Vietnam	Genbank	C4	I
KJ686182	2011	Vietnam	Genbank	C4	I
KJ686185	2011	Vietnam	Genbank	C4	I
KJ686189	2011	Vietnam	Genbank	C4	I
KJ686194	2012	Vietnam	Genbank	C4	I
KJ686196	2012	Vietnam	Genbank	C4	I
KJ686199	2012	Vietnam	Genbank	C4	I
KJ686200	2011	Vietnam	Genbank	C4	I
KJ686202	2012	Vietnam	Genbank	C4	I
KJ686205	2012	Vietnam	Genbank	C4	I
KJ686209	2011	Vietnam	Genbank	C4	I
KJ686210	2012	Vietnam	Genbank	C4	I
KJ686216	2012	Vietnam	Genbank	C4	I
KJ686218	2011	Vietnam	Genbank	C4	I
KJ686226	2012	Vietnam	Genbank	C4	I
KJ686228	2011	Vietnam	Genbank	C4	I
KJ686233	2011	Vietnam	Genbank	C4	I
KJ686239	2011	Vietnam	Genbank	C4	I
KJ686243	2012	Vietnam	Genbank	C4	I
KJ686245	2012	Vietnam	Genbank	C4	I
KJ686250	2012	Vietnam	Genbank	C4	I

KJ686253	2012	Vietnam	Genbank	C4	I
KJ686257	2011	Vietnam	Genbank	C4	I
KJ686259	2012	Vietnam	Genbank	C4	I
KJ686267	2012	Vietnam	Genbank	C4	I
KJ686272	2011	Vietnam	Genbank	C4	I
KJ686274	2012	Vietnam	Genbank	C4	I
KJ686278	2011	Vietnam	Genbank	C4	I
KJ686284	2011	Vietnam	Genbank	C4	I
KJ686285	2012	Vietnam	Genbank	C4	I
KJ686289	2012	Vietnam	Genbank	C4	I
KJ686290	2011	Vietnam	Genbank	C4	I
KJ686295	2012	Vietnam	Genbank	C4	I
KJ686299	2011	Vietnam	Genbank	C4	I
KJ686301	2011	Vietnam	Genbank	C4	I
KJ784495	2011	China	Genbank	C4	O
KM211580	2012	China	Genbank	C4	I
KP198623	2012	China	Genbank	C4	I
KP266579	2006	China	Genbank	C4	I
KP274875	2011	China	Genbank	C4	I
KP289421	2013	China	Genbank	C4	I
KP308401	2012	Cambodia	Genbank	C4	I
KP308402	2012	Cambodia	Genbank	C4	I
KP308403	2012	Cambodia	Genbank	C4	I
KP308405	2012	Cambodia	Genbank	C4	I
KP308407	2012	Cambodia	Genbank	C4	I
KP308408	2012	Cambodia	Genbank	C4	I
KP308409	2012	Cambodia	Genbank	C4	I
KP308412	2012	Cambodia	Genbank	C4	I
KP308414	2012	Cambodia	Genbank	C4	I
KP308415	2012	Cambodia	Genbank	C4	I
KP308418	2012	Cambodia	Genbank	C4	I
KP308419	2012	Cambodia	Genbank	C4	I
KP308421	2012	Cambodia	Genbank	C4	I
KP308422	2012	Cambodia	Genbank	C4	I
KP308423	2012	Cambodia	Genbank	C4	I
KP308424	2012	Cambodia	Genbank	C4	I
KP308428	2012	Cambodia	Genbank	C4	I
KP308429	2012	Cambodia	Genbank	C4	I
KP308431	2013	Cambodia	Genbank	C4	I
KP308432	2012	Cambodia	Genbank	C4	I
KP308433	2012	Cambodia	Genbank	C4	I
KP308434	2012	Cambodia	Genbank	C4	I
KP308436	2012	Cambodia	Genbank	C4	I
KP308437	2012	Cambodia	Genbank	C4	I

KP308438	2012	Cambodia	Genbank	C4	I
KP308439	2012	Cambodia	Genbank	C4	I
KP308440	2012	Cambodia	Genbank	C4	I
KP308441	2012	Cambodia	Genbank	C4	I
KP308442	2012	Cambodia	Genbank	C4	I
KP308443	2012	Cambodia	Genbank	C4	I
KP308444	2012	Cambodia	Genbank	C4	I
KP308449	2012	Cambodia	Genbank	C4	I
KP308450	2012	Cambodia	Genbank	C4	N
KP308451	2012	Cambodia	Genbank	C4	I
KP308452	2012	Cambodia	Genbank	C4	I
KP308453	2012	Cambodia	Genbank	C4	I
KP308457	2012	Cambodia	Genbank	C4	I
KP308458	2013	Cambodia	Genbank	C4	I
KP308459	2012	Cambodia	Genbank	C4	I
KT428646	2014	China	Genbank	C4	I
KU254595	2014	China	Genbank	C4	I
KX197455	2012	Cambodia	Genbank	C4	I
KX197456	2012	Cambodia	Genbank	C4	I
KX197457	2012	Cambodia	Genbank	C4	I
KX197458	2012	Cambodia	Genbank	C4	I
KX197460	2012	Cambodia	Genbank	C4	I
KX197461	2012	Cambodia	Genbank	C4	I
KX197462	2012	Cambodia	Genbank	C4	I
KX197463	2012	Cambodia	Genbank	C4	I
KX197464	2012	Cambodia	Genbank	C4	I
KX197465	2012	Cambodia	Genbank	C4	I
KX893543	2015	China	Genbank	C4	I
KY315729	2015	China	Genbank	C4	I
MF431793	2013	China	Genbank	C4	I
MG756693	2015	China	Genbank	C4	I
MG756696	2005	China	Genbank	C4	I
MG756701	2005	China	Genbank	C4	I
MG756715	2010	China	Genbank	C4	I
MG756720	2011	China	Genbank	C4	I
MG756724	2012	China	Genbank	C4	I
MG756725	2012	China	Genbank	C4	I
MG773123	2014	China	Genbank	C4	I
HB/SC39	2019	China	This study	C4	I
SD/WF305	2019	China	This study	C4	I

Supplementary Table S7. Information on 234 China C4 for phylogeographic analysis.

Strain name/Genbank No.	Isolated province	Isolated year	Isolated region	Source	Genotype/subgenotype	Evolutionary lineage
GX/000601	Guangxi	2017	South China	This study	C4	I
LN/11-11	Liaoning	2017	Northeast China	This study	C4	I
TJ/116	Tianjing	2016	North China	This study	C4	I
GD/122	Guangdong	2017	South China	This study	C4	I
XJ/13	Xinjiang	2018	Northwest China	This study	C4	I
GS/133	Gansu	2019	Northwest China	This study	C4	I
SX/164	Shanxi	2017	North China	This study	C4	I
GS/165	Gansu	2018	Northwest China	This study	C4	I
BJ/17	Beijing	2017	North China	This study	C4	I
SC/17	Sichuan	2016	Southwest China	This study	C4	I
SX/18	Shanxi	2018	North China	This study	C4	I
HB/187	Hebei	2017	North China	This study	C4	I
BJ/19	Beijing	2018	North China	This study	C4	I
HLJ/19	Heilongjiang	2017	Northeast China	This study	C4	I
HB/195	Hebei	2016	North China	This study	C4	I
AH/2	Anhui	2016	East China	This study	C4	I
CQ/20	Chongqing	2017	Southwest China	This study	C4	I
JL/20	Jilin	2018	Northeast China	This study	C4	I
JL/21	Jilin	2018	Northeast China	This study	C4	I
JX/21	Jiangxi	2017	East China	This study	C4	I
BJ/22	Beijing	2017	North China	This study	C4	I
GD/22	Guangdong	2018	South China	This study	C4	I
JL/23	Jilin	2018	Northeast China	This study	C4	I
HB/232	Hebei	2016	North China	This study	C4	I
HaN/24	Hainan	2018	South China	This study	C4	I
HB/24	Hebei	2018	North China	This study	C4	I
SC/24	Sichuan	2016	Southwest China	This study	C4	I
HeN/250	Henan	2018	Central China	This study	C4	I
JX/26	Jiangxi	2018	East China	This study	C4	I
SaX/26	Shaanxi	2017	Northwest China	This study	C4	I
LN/26-53	Liaoning	2017	Northeast China	This study	C4	I
LN/26-67	Liaoning	2017	Northeast China	This study	C4	I
HB/269	Hebei	2018	North China	This study	C4	I
HeN/276	Henan	2017	Central China	This study	C4	I
HeN/287	Henan	2017	Central China	This study	C4	I
GS/3	Gansu	2016	Northwest China	This study	C4	I
TJ/3	Tianjing	2018	North China	This study	C4	I
HeN/313	Henan	2017	Central China	This study	C4	I
TJ/320	Tianjing	2016	North China	This study	C4	I
HLJ/34	Heilongjiang	2017	Northeast China	This study	C4	I
HaN/35	Hainan	2018	South China	This study	C4	I
SaX/35	Shaanxi	2017	Northwest China	This study	C4	I

GS/375	Gansu	2017	Northwest China	This study	C4	I
HuN/39	Hunan	2016	Central China	This study	C4	I
CQ/4	Chongqing	2017	Southwest China	This study	C4	I
GS/41	Gansu	2016	Northwest China	This study	C4	I
QH/44	Qinghai	2017	Northwest China	This study	C4	I
HaN/5	Hainan	2017	South China	This study	C4	I
CQ/52	Chongqing	2016	Southwest China	This study	C4	I
TJ/53	Tianjing	2017	North China	This study	C4	I
GD/57	Guangdong	2018	South China	This study	C4	I
JX/59	Jiangxi	2016	East China	This study	C4	I
HaN/6	Hainan	2017	South China	This study	C4	I
QH/63	Qinghai	2017	Northwest China	This study	C4	I
AH/64	Anhui	2017	East China	This study	C4	I
HLJ/66	Heilongjiang	2018	Northeast China	This study	C4	I
HLJ/67	Heilongjiang	2018	Northeast China	This study	C4	I
XJ/70	Xinjiang	2016	Northwest China	This study	C4	I
HLJ/71	Heilongjiang	2018	Northeast China	This study	C4	I
HuN/71	Hunan	2016	Central China	This study	C4	I
JX/76	Jiangxi	2016	East China	This study	C4	I
XJ/78	Xinjiang	2016	Northwest China	This study	C4	I
HLJ/79	Heilongjiang	2018	Northeast China	This study	C4	I
GD/8	Guangdong	2017	South China	This study	C4	I
HLJ/80	Heilongjiang	2018	Northeast China	This study	C4	I
JL/82	Jilin	2018	Northeast China	This study	C4	I
JL/83	Jilin	2018	Northeast China	This study	C4	I
XJ/83	Xinjiang	2016	Northwest China	This study	C4	I
BJ/9	Beijing	2018	North China	This study	C4	I
GS/9	Gansu	2016	Northwest China	This study	C4	I
XJ/93	Xinjiang	2016	Northwest China	This study	C4	I
HeN/a4	Henan	2018	Central China	This study	C4	I
HeN/a5	Henan	2018	Central China	This study	C4	I
YN/A50	Yunnan	2017	Southwest China	This study	C4	I
YN/A80	Yunnan	2018	Southwest China	This study	C4	I
YN/A96	Yunnan	2016	Southwest China	This study	C4	I
YN/B89	Yunnan	2017	Southwest China	This study	C4	I
YN/C76	Yunnan	2018	Southwest China	This study	C4	I
YN/C99	Yunnan	2018	Southwest China	This study	C4	I
EU131776	Taiwan	2002	Taiwan of China	Genbank	C4	I
EU812515	Anhui	2008	East China	Genbank	C4	I
YN/F257	Yunnan	2016	Southwest China	This study	C4	I
HuN/FC47	Hunan	2016	Central China	This study	C4	I
FJ194964	Guangdong	2008	South China	Genbank	C4	I
FJ360544	Guizhou	2008	Southwest China	Genbank	C4	I
FJ360545	Guizhou	2008	Southwest China	Genbank	C4	I

FJ439769	Anhui	2008	East China	Genbank	C4	I
FJ606449	Beijing	2008	North China	Genbank	C4	I
FJ606450	Beijing	2008	North China	Genbank	C4	N
FJ607335	Guangdong	2008	South China	Genbank	C4	I
FJ607337	Guangdong	2008	South China	Genbank	C4	N
GQ231926	Taiwan	2005	Taiwan of China	Genbank	C4	I
GQ231928	Taiwan	2004	Taiwan of China	Genbank	C4	I
GQ231930	Taiwan	2004	Taiwan of China	Genbank	C4	I
GQ231931	Taiwan	2004	Taiwan of China	Genbank	C4	I
GQ231932	Taiwan	2004	Taiwan of China	Genbank	C4	I
GQ231933	Taiwan	2008	Taiwan of China	Genbank	C4	I
GQ231937	Taiwan	2005	Taiwan of China	Genbank	C4	I
GQ231940	Taiwan	2004	Taiwan of China	Genbank	C4	I
GQ279369	HongKong	2008	South China	Genbank	C4	I
GQ892830	Guangxi	2008	South China	Genbank	C4	I
GQ994988	Anhui	2009	East China	Genbank	C4	I
GQ994989	Chongqing	2009	Southwest China	Genbank	C4	I
GQ994990	Chongqing	2009	Southwest China	Genbank	C4	I
GQ994991	Chongqing	2009	Southwest China	Genbank	C4	I
GQ994992	Henan	2009	Central China	Genbank	C4	I
GU198369	Anhui	2008	East China	Genbank	C4	I
GU396280	Gansu	2009	Northwest China	Genbank	C4	I
GU459071	Anhui	2008	East China	Genbank	C4	I
NX/GY038	Ningxia	2017	Northwest China	This study	C4	I
HM002485	Beijing	2008	North China	Genbank	C4	I
HM002489	Beijing	2009	North China	Genbank	C4	I
HM053669	Beijing	2008	North China	Genbank	C4	I
HM053670	Beijing	2009	North China	Genbank	C4	I
HM245927	Henan	2010	Central China	Genbank	C4	I
HQ129932	Beijing	2006	North China	Genbank	C4	I
HQ407557	Liaoning	2010	Northeast China	Genbank	C4	I
HQ423142	Yunnan	2009	Southwest China	Genbank	C4	O
HQ423143	Yunnan	2009	Southwest China	Genbank	C4	O
HQ694985	Anhui	2008	East China	Genbank	C4	I
HQ694986	Anhui	2010	East China	Genbank	C4	I
HQ850973	Fujian	2010	East China	Genbank	C4	I
HQ998852	Henan	2009	Central China	Genbank	C4	I
YN/I12	Yunnan	2019	Southwest China	This study	C4	I
YN/I13	Yunnan	2016	Southwest China	This study	C4	I
YN/J48	Yunnan	2017	Southwest China	This study	C4	I
JF820313	Beijing	2010	North China	Genbank	C4	I
JF820314	Beijing	2010	North China	Genbank	C4	I
JF894381	Beijing	2011	North China	Genbank	C4	I
JF894382	Beijing	2011	North China	Genbank	C4	I

JF894383	Beijing	2011	North China	Genbank	C4	I
JN052925	Henan	2011	Central China	Genbank	C4	I
JN230523	Hubei	2009	Central China	Genbank	C4	I
JN256060	Henan	2009	Central China	Genbank	C4	I
JQ086366	Zhejiang	2011	East China	Genbank	C4	I
JQ517316	Henan	2010	Central China	Genbank	C4	I
JQ681218	Henan	2008	Central China	Genbank	C4	I
JQ708209	Beijing	2010	North China	Genbank	C4	I
JQ708210	Beijing	2009	North China	Genbank	C4	I
JQ742001	Guangxi	2001	South China	Genbank	C4	I
JQ742002	Guangxi	2001	South China	Genbank	C4	I
JQ804832	Hubei	2010	Central China	Genbank	C4	I
JX111888	Guangdong	2011	South China	Genbank	C4	I
JX111889	Guangdong	2011	South China	Genbank	C4	I
JX111891	Guangdong	2011	South China	Genbank	C4	I
HLJ/JX114	Heilongjiang	2016	Northeast China	This study	C4	I
HLJ/JX119	Heilongjiang	2016	Northeast China	This study	C4	I
JX244183	Shandong	2009	East China	Genbank	C4	I
JX678874	Chongqing	2003	Southwest China	Genbank	C4	I
JX678877	Anhui	2008	East China	Genbank	C4	I
JX678882	Shandong	2007	East China	Genbank	C4	I
JX678883	Shandong	2009	East China	Genbank	C4	I
JX678885	Shanghai	2002	East China	Genbank	C4	I
JX678886	Shanghai	2002	East China	Genbank	C4	I
HLJ/JX77	Heilongjiang	2016	Northeast China	This study	C4	I
HLJ/JX80	Heilongjiang	2016	Northeast China	This study	C4	I
JX986737	Hubei	2011	Central China	Genbank	C4	I
JX986738	Hubei	2011	Central China	Genbank	C4	I
KC414134	Jilin	2011	Northeast China	Genbank	C4	O
KC436265	HongKong	2004	South China	Genbank	C4	I
KC436267	HongKong	2006	South China	Genbank	C4	I
KC436268	HongKong	2007	South China	Genbank	C4	I
KC436269	HongKong	2008	South China	Genbank	C4	I
KC436271	HongKong	2009	South China	Genbank	C4	I
KC436272	HongKong	2010	South China	Genbank	C4	I
KC570452	Shanghai	2012	East China	Genbank	C4	I
KC954662	Anhui	2007	East China	Genbank	C4	I
KF142411	Hunan	2013	Central China	Genbank	C4	I
KF142412	Hunan	2013	Central China	Genbank	C4	I
KF142413	Hunan	2013	Central China	Genbank	C4	I
KF826491	Jiangsu	2012	East China	Genbank	C4	I
KJ004552	Hunan	2011	Central China	Genbank	C4	I
KJ004554	Hunan	2012	Central China	Genbank	C4	I
KJ004556	Ningxia	2010	Northwest China	Genbank	C4	I

KJ004557	Ningxia	2010	Northwest China	Genbank	C4	I
KJ004559	Guangdong	2010	South China	Genbank	C4	I
KJ004560	Guangdong	2010	South China	Genbank	C4	I
KJ186974	Taiwan	2011	Taiwan of China	Genbank	C4	I
KJ508182	Hubei	2012	Central China	Genbank	C4	I
KJ632498	Zhejiang	2011	East China	Genbank	C4	I
KJ784495	Zhejiang	2011	East China	Genbank	C4	O
KM211580	Henan	2012	Central China	Genbank	C4	I
KM591215	Hubei	2010	Central China	Genbank	C4	I
KM593929	Taiwan	2011	Taiwan of China	Genbank	C4	I
KP198623	Hubei	2012	Central China	Genbank	C4	I
KP266579	Jiangsu	2006	East China	Genbank	C4	I
KP274875	Taiwan	2011	Taiwan of China	Genbank	C4	I
KP289421	Zhejiang	2013	East China	Genbank	C4	I
KP289428	Zhejiang	2013	East China	Genbank	C4	I
KT428644	Guangdong	2014	South China	Genbank	C4	I
KT428646	Guangdong	2014	South China	Genbank	C4	I
KT428649	Guangdong	2014	South China	Genbank	C4	I
KU254595	Beijing	2014	North China	Genbank	C4	I
KU254596	Beijing	2014	North China	Genbank	C4	I
KX893543	Yunnan	2015	Southwest China	Genbank	C4	I
KY315729	Shandong	2015	East China	Genbank	C4	I
KY425527	Yunnan	2013	Southwest China	Genbank	C4	I
KY612315	Jiangsu	2015	East China	Genbank	C4	I
MF362981	Guangdong	2010	South China	Genbank	C4	I
MF431793	Guangdong	2013	South China	Genbank	C4	I
MG520666	Jiangsu	2017	East China	Genbank	C4	I
MG756691	Taiwan	2016	Taiwan of China	Genbank	C4	I
MG756692	Taiwan	2016	Taiwan of China	Genbank	C4	I
MG756693	Taiwan	2015	Taiwan of China	Genbank	C4	I
MG756696	Taiwan	2005	Taiwan of China	Genbank	C4	I
MG756699	Taiwan	2005	Taiwan of China	Genbank	C4	I
MG756701	Taiwan	2005	Taiwan of China	Genbank	C4	I
MG756703	Taiwan	2005	Taiwan of China	Genbank	C4	I
MG756715	Taiwan	2010	Taiwan of China	Genbank	C4	I
MG756716	Taiwan	2010	Taiwan of China	Genbank	C4	I
MG756717	Taiwan	2010	Taiwan of China	Genbank	C4	I
MG756719	Taiwan	2010	Taiwan of China	Genbank	C4	I
MG756720	Taiwan	2011	Taiwan of China	Genbank	C4	I
MG756722	Taiwan	2011	Taiwan of China	Genbank	C4	I
MG756724	Taiwan	2012	Taiwan of China	Genbank	C4	I
MG756725	Taiwan	2012	Taiwan of China	Genbank	C4	I
MG773123	Anhui	2014	East China	Genbank	C4	I
MG773125	Anhui	2014	East China	Genbank	C4	I

YN/P35	Yunnan	2019	Southwest China	This study	C4	I
YN/P53	Yunnan	2019	Southwest China	This study	C4	I
GS/SC158	Gansu	2019	Northwest China	This study	C4	I
GS/SC291	Gansu	2019	Northwest China	This study	C4	I
HB/SC353	Hebei	2017	North China	This study	C4	I
HB/SC39	Hebei	2019	North China	This study	C4	I
GS/SC476	Gansu	2017	Northwest China	This study	C4	I
GS/SC477	Gansu	2017	Northwest China	This study	C4	I
GS/SC576	Gansu	2018	Northwest China	This study	C4	I
GS/SC708	Gansu	2018	Northwest China	This study	C4	I
YN/SCF276	Yunnan	2016	Southwest China	This study	C4	I
SD/WF305	Shandong	2019	East China	This study	C4	I
NX/WZ90	Ningxia	2018	Northwest China	This study	C4	I
NX/WZ94	Ningxia	2018	Northwest China	This study	C4	I
CQ/Y86	Chongqing	2016	Southwest China	This study	C4	I
NX/YC188	Ningxia	2018	Northwest China	This study	C4	I

Supplementary Table S8. Marginal likelihood estimation of the molecular clock model and coalescent model for 256 global EV-A71 sequences. The best-fitting model combination was highlighted in bolded font.

Molecular clock model	Coalescent tree prior	PS	SS
Strict clock	Bayesian SkyGrid	-17209.8	-17248.0
Strict clock	Bayesian Skyline	-17196.3	-17246.8
Strict clock	Constant Size	-17218.3	-17254.0
Strict clock	Exponential Growth	-17205.5	-17228.1
Strict clock	GMRF Bayesian Skyride	-17293.9	-17341.2
Uncorrelated relaxed clock(exponential)	Bayesian SkyGrid	-17257.2	-17303.5
Uncorrelated relaxed clock(exponential)	Bayesian Skyline	-17243.0	-17291.6
Uncorrelated relaxed clock(exponential)	Constant Size	-17268.4	-17293.7
Uncorrelated relaxed clock(exponential)	Exponential Growth	-17256.4	-17295.4
Uncorrelated relaxed clock(exponential)	GMRF Bayesian Skyride	-17333.4	-17366.5
Uncorrelated relaxed clock(lognormal)	Bayesian SkyGrid	-17179.1	-17221.8
Uncorrelated relaxed clock(lognormal)	Bayesian Skyline	-17183.1	-17232.7
**Uncorrelated relaxed clock(lognormal)**	**Constant Size**	**-17182.2**	**-17199.6**
Uncorrelated relaxed clock(lognormal)	Exponential Growth	-17185.8	-17216.8
Uncorrelated relaxed clock(lognormal)	GMRF Bayesian Skyride	-17255.4	-17312.0

Supplementary Table S9. Marginal likelihood estimation of the molecular clock model and coalescent model for 85 Asian B5 sequences. The best-fitting model combination was highlighted in bolded font.

Molecular clock model	Coalescent tree prior	PS	SS
Strict clock	Bayesian SkyGrid	-3937.4	-3935.7
Strict clock	Bayesian Skyline	-3929.6	-3928.7
Strict clock	Constant Size	-3935.7	-3932.3
Strict clock	Exponential Growth	-3931.0	-3929.3
Strict clock	GMRF Bayesian Skyride	-3946.4	-3944.8
Uncorrelated relaxed clock(exponential)	Bayesian SkyGrid	-3943.3	-3939.8
Uncorrelated relaxed clock(exponential)	Bayesian Skyline	-3941.1	-3936.2
Uncorrelated relaxed clock(exponential)	Constant Size	-3942.6	-3938.6
Uncorrelated relaxed clock(exponential)	Exponential Growth	-3943.3	-3939.8
Uncorrelated relaxed clock(exponential)	GMRF Bayesian Skyride	-3949.2	-3945.9
Uncorrelated relaxed clock(lognormal)	Bayesian SkyGrid	-3933.7	-3932.8
**Uncorrelated relaxed clock(lognormal)**	**Bayesian Skyline**	**-3928.2**	**-3926.4**
Uncorrelated relaxed clock(lognormal)	Constant Size	-3935.7	-3927.6
Uncorrelated relaxed clock(lognormal)	Exponential Growth	-3929.1	-3928.6
Uncorrelated relaxed clock(lognormal)	GMRF Bayesian Skyride	-3943.3	-3944.6

Supplementary Table S10. Marginal likelihood estimation of the molecular clock model and coalescent model for 170 Asian C4 sequences. The best-fitting model combination was highlighted in bolded font.

Molecular clock model	Coalescent tree prior	PS	SS
Strict clock	Bayesian SkyGrid	-8529.3	-8533.7
**Strict clock**	**Bayesian Skyline**	**-8505.3**	**-8508.7**
Strict clock	Constant Size	-8544.3	-8550.9
Strict clock	Exponential Growth	-8524.8	-8525.3
Strict clock	GMRF Bayesian Skyride	-8556.9	-8565.3
Uncorrelated relaxed clock(exponential)	Bayesian SkyGrid	-8563.0	-8571.2
Uncorrelated relaxed clock(exponential)	Bayesian Skyline	-8543.8	-8553.5
Uncorrelated relaxed clock(exponential)	Constant Size	-8556.1	-8553.9
Uncorrelated relaxed clock(exponential)	Exponential Growth	-8551.5	-8560.8
Uncorrelated relaxed clock(exponential)	GMRF Bayesian Skyride	-8579.2	-8579.2
Uncorrelated relaxed clock(lognormal)	Bayesian SkyGrid	-8540.2	-8542.6
Uncorrelated relaxed clock(lognormal)	Bayesian Skyline	-8506.3	-8514.7
Uncorrelated relaxed clock(lognormal)	Constant Size	-8539.0	-8531.6
Uncorrelated relaxed clock(lognormal)	Exponential Growth	-8524.2	-8533.3
Uncorrelated relaxed clock(lognormal)	GMRF Bayesian Skyride	-8551.0	-8556.2

Supplementary Table S11. Marginal likelihood estimation of the molecular clock model and coalescent model for 234 China C4 sequences. The best-fitting model combination was highlighted in bolded font.

Molecular clock model	Coalescent tree prior	PS	SS
Strict clock	Bayesian SkyGrid	-11909.5	-11928.1
Strict clock	Bayesian Skyline	-11898.4	-11919.4
Strict clock	Constant Size	-11908.5	-11930.5
Strict clock	Exponential Growth	-11897.1	-11921.8
Strict clock	GMRF Bayesian Skyride	-11923.8	-11934.8
Uncorrelated relaxed clock(exponential)	Bayesian SkyGrid	-11917.7	-11937.2
Uncorrelated relaxed clock(exponential)	Bayesian Skyline	-11910.9	-11939.2
Uncorrelated relaxed clock(exponential)	Constant Size	-11923.0	-11949.8
Uncorrelated relaxed clock(exponential)	Exponential Growth	-11913.3	-11938.7
Uncorrelated relaxed clock(exponential)	GMRF Bayesian Skyride	-11928.3	-11954.0
Uncorrelated relaxed clock(lognormal)	Bayesian SkyGrid	-11891.6	-11917.0
Uncorrelated relaxed clock(lognormal)	Bayesian Skyline	-11872.4	-11894.1
Uncorrelated relaxed clock(lognormal)	Constant Size	-11892.5	-11901.9
**Uncorrelated relaxed clock(lognormal)**	**Exponential Growth**	**-11873.3**	**-11889.6**
Uncorrelated relaxed clock(lognormal)	GMRF Bayesian Skyride	-11897.9	-11926.5

Supplementary table S12. Analysis of the spatial structure of C4 strains in Asia.

Statistic	observed mean (95% HPD CIs)[d]	null mean (95% HPD CIs)	*P*-value
AI[a]	1.18(0.71, 1.67)	12.25(10.60, 13.61)	<0.001
PS[b]	12.93(12.00, 14.00)	79.34(72.73, 84.25)	<0.001
MC[c](Cambodia)	15.35(15.00, 17.00)	3.37(2.45, 5.00)	0.01
MC (China)	16.42(12.00, 19.00)	2.70(2.05, 4.05)	0.01
MC (Vietnam)	29.16(29.00, 31.00)	2.57(2.04, 3.24)	0.01

[a]AI, association index.

[b]PS, parsimony score.

[c]MC, maximum monophyletic clade.

[d]HPD CIs, highest posterior density confidence intervals.

Supplementary Table S13. Statistically supported migration rates of Asian B5 based on the *VP1* region.

From	To	Mean migration rate	Bayes factor (BF)[a]	Indicator
Taiwan of China	Vietnam	3.04	11050.89	1.00
Thailand	Taiwan of China	2.60	2761.80	1.00
Vietnam	Thailand	1.60	46.21	0.97

[a]The BF value > 3 and indicator > 0.50 of statistically supported migration rates are shown.

Supplementary Table S14. Statistically supported migration rates of Asian C4 based on the *VP1* region.

From	To	Mean migration rate	BF[a]	Indicator
Vietnam	Cambodia	9.40	11050.89	1.00
China	Vietnam	3.33	219.81	0.99

[a]BF, Bayes factor. The BF value > 3 and indicator > 0.50 of statistically supported migration rates are shown.

Supplementary table S15. Analysis of the spatial structure of C4 strains in China.

Statistic	observed mean (95% HPD CIs)[d]	null mean (95% HPD CIs)	*P*-value
AI[a]	15.64(14.42, 16.83)	22.14(20.97, 23.33)	<0.001
PS[b]	128.65(125.00, 132.00)	162.38(156.09, 168.22)	<0.001
MC[c] (Central China)	2.24(2.00, 3.00)	1.79(1.03, 2.99)	0.36
MC (East China)	5.05(5.00, 6.00)	1.47(1.00, 2.05)	0.01
MC (North China)	3.09(3.00, 4.00)	1.93(1.37, 2.61)	0.04
MC (Northeast China)	2.26(2.00, 3.00)	1.75(1.02, 3.00)	0.33
MC (Northwest China)	2.00(2.00, 2.00)	1.83(1.05, 3.00)	0.40
MC (South China)	2.17(2.00, 3.00)	1.91(1.16, 2.70)	0.46
MC (Southwest China)	1.79(1.00, 2.00)	1.74(1.05, 2.27)	0.37
MC (Taiwan of China)	2.90(2.00, 4.00)	1.69(1.02, 2.24)	0.03

[a]AI, association index.

[b]PS, parsimony score.

[c]MC, maximum monophyletic clade.

[d]HPD CIs, highest posterior density confidence intervals.

Supplementary Table S16. Statistically supported migration rates of China C4 based on the *VP1* region.

From	To	Mean migration rate	BF[a]	Indicator
East China	North China	17.02	56512.85	1.00
East China	South China	21.39	56512.85	1.00
East China	Central China	22.04	56512.85	1.00
East China	Taiwan of China	6.88	2254.49	1.00
East China	Southwest China	16.90	431.85	0.99
North China	Northeast China	6.84	56512.85	1.00
North China	Northwest China	12.53	1816.92	1.00
North China	East China	5.78	37.36	0.86
North China	Central China	3.11	21.85	0.78
North China	South China	2.72	12.32	0.66
South China	Northwest China	1.44	7.58	0.55
Southwest China	North China	3.11	23.85	0.79
Southwest China	Northeast China	1.66	20.72	0.77

[a]BF, Bayes factor. The BF value > 3 and indicator > 0.50 of statistically supported migration rates are shown.

Supplementary Table S17. Selective pressures in *VP1* and *3D*^{pol} region of lineage H (contains two subgenotypes B4 and B5), C1(contains two lineages -E, -Q), C2(contains two lineages -E, -M).

Subgenotype(Lineage)	N[a]	*VP1*				*3D*^{pol}			
		SLAC		MEME		SLAC		MEME	
		dN/dS	Positively selection site (amino acid position)	dN/dS	Positively selection site (amino acid position)	dN/dS	Positively selection site (amino acid position)	dN/dS	Positively selection site (amino acid position)
B4(H)	6	0.0653	NA	0.0583	NA	0.0648	NA	0.0546	NA
B5(H)	142	0.0605	145	0.0534	98	0.0703	NA	0.0597	383
C1(E)	12	0.0090	NA	0.0082	NA	0.0521	NA	0.0464	NA
C1(Q)	14	0.0458	NA	0.0430	NA	0.0785	NA	0.0682	NA
C2(E)	32	0.0241	NA	0.0224	NA	0.0584	NA	0.0513	173
C2(M)	3	0.4920	NA	0.4800	NA	0.3730	NA	0.3250	NA

[a]The number of sequences analyzed in each dataset.

Fig. S1

Geographic and temporal distribution of the 200 newly sequenced EV-A71. The horizontal coordinate is the geographic distribution and the vertical coordinate is the number of sequences, with the years indicated by different colors.

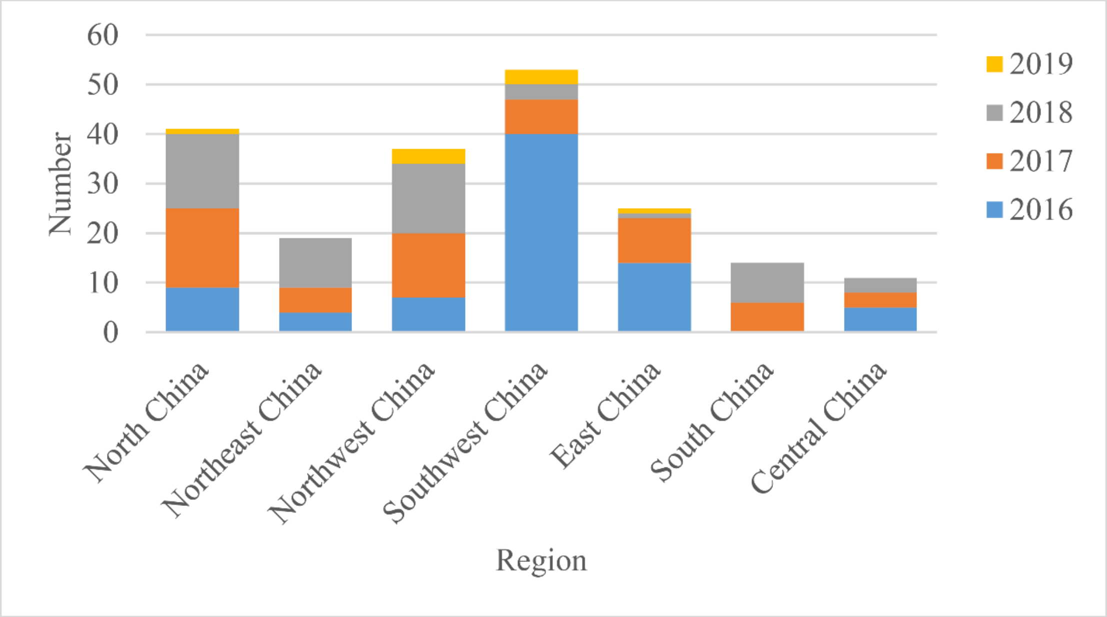

Fig. S2

Selection of 259 representative sequences. A neighbor-joining tree was constructed based on the ORF of 972 global EV-A71 sequences, and 259 representative sequences were selected from them for phylogenetic analysis.

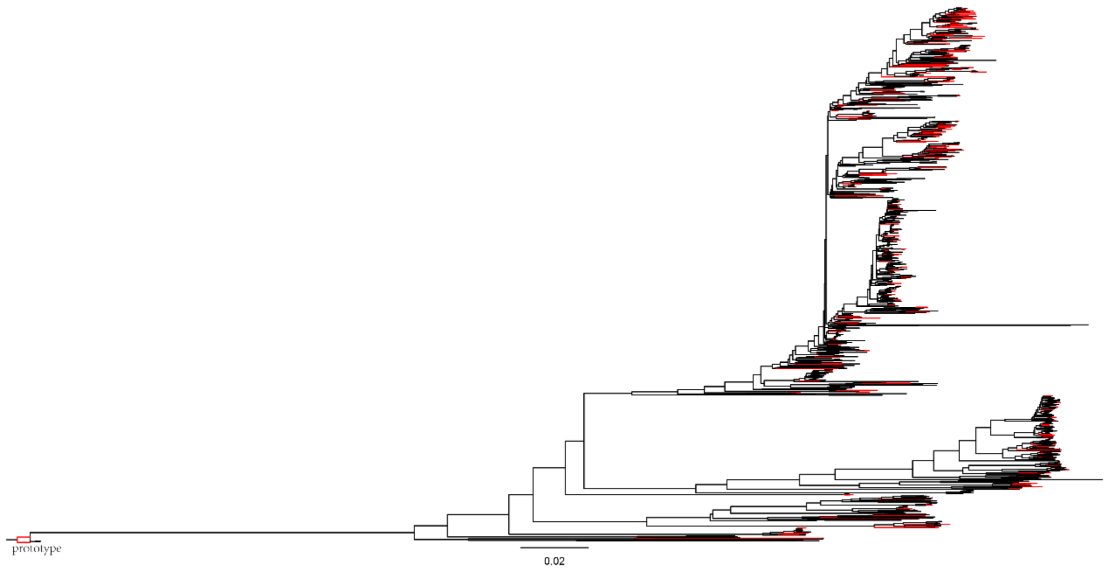

Fig. S3

The results of root-to-tip regression analysis and date-randomization test (DRT) in the *VP1* region of the datasets sequence. Both show results from root-to-tip regression analysis on the left, and DRT results on the right. Only if the 95% credibility interval of the rate estimate does not fall within the 95% credibility intervals of the rate estimates from the date-randomized replicates， the dataset is considered to have sufficient temporal signal for molecular dating(2, 3).A)256 global *VP1* sequences were used for temporal dynamics analysis; B)85 Asian *VP1* sequences were used for phytogeographic analysis; C)170 Asian *VP1* sequences were used for phytogeographic analysis; D)234 China *VP1* sequences were used for phytogeographic analysis.

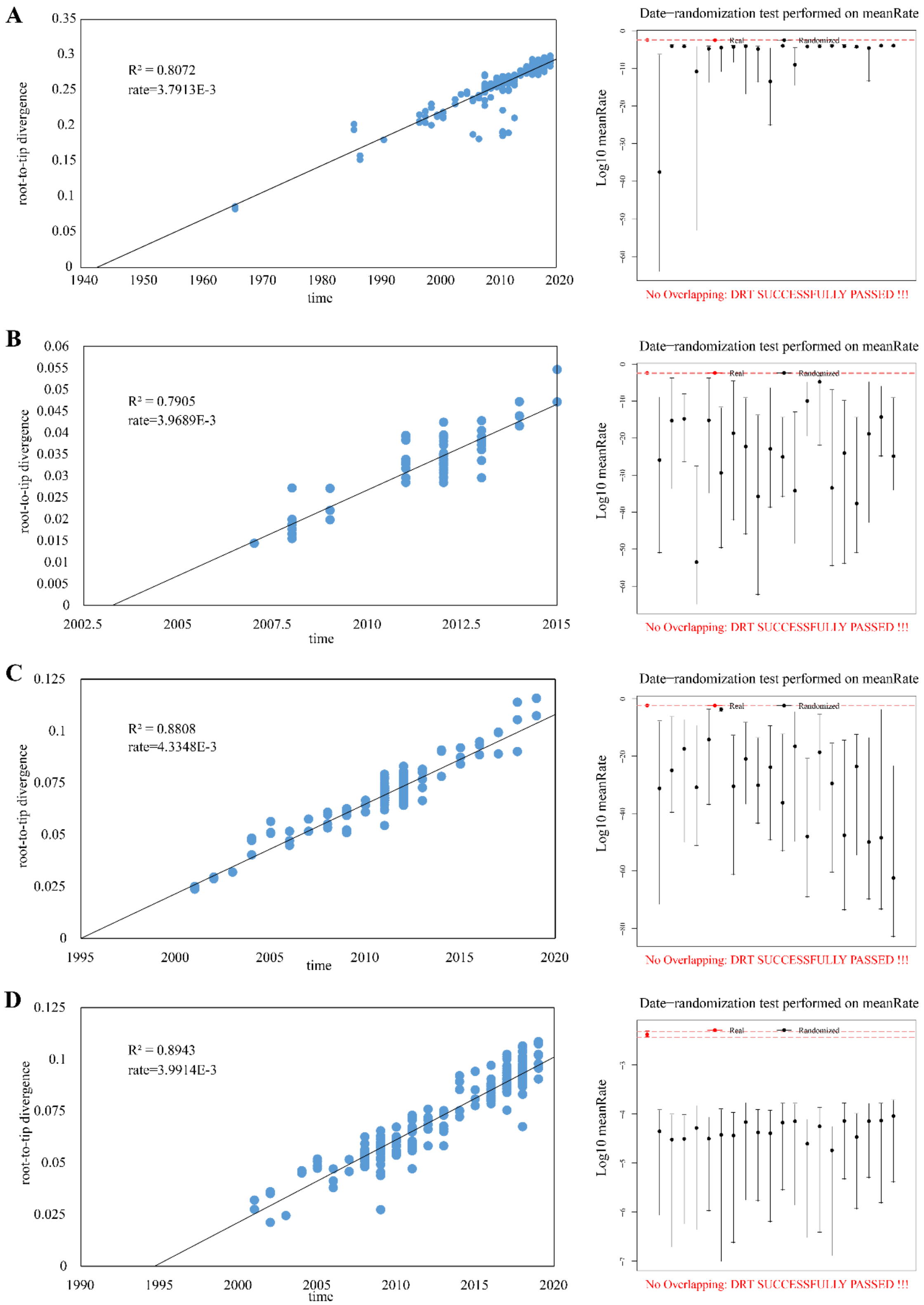
A
R² = 0.8072
rate=3.7913E-3
root-to-tip divergence
time
Date−randomization test performed on meanRate
Log10 meanRate
Real
Randomized
No Overlapping: DRT SUCCESSFULLY PASSED !!!
B
R² = 0.7905
rate=3.9689E-3
root-to-tip divergence
time
Date−randomization test performed on meanRate
Log10 meanRate
Real
Randomized
No Overlapping: DRT SUCCESSFULLY PASSED !!!
C
R² = 0.8808
rate=4.3348E-3
root-to-tip divergence
time
Date−randomization test performed on meanRate
Log10 meanRate
Real
Randomized
No Overlapping: DRT SUCCESSFULLY PASSED !!!
D
R² = 0.8943
rate=3.9914E-3
root-to-tip divergence
time
Date−randomization test performed on meanRate
Log10 meanRate
Real
Randomized
No Overlapping: DRT SUCCESSFULLY PASSED !!!

Fig. S4

The maximum likelihood tree of 259 representative sequences based on the ORF region. The maximum likelihood tree was constructed based on the ORF of 259 EV-A71 representative sequences. The sequences of the same clade are represented by triangles, and their size is proportional to the number of sequences in each clade. A) The *VP1* region of the sequences belonging to the same genotype/subgenotype filled with the same color. B) The $3D^{pol}$ region of the sequences belonging to the same lineage filled with the same color.

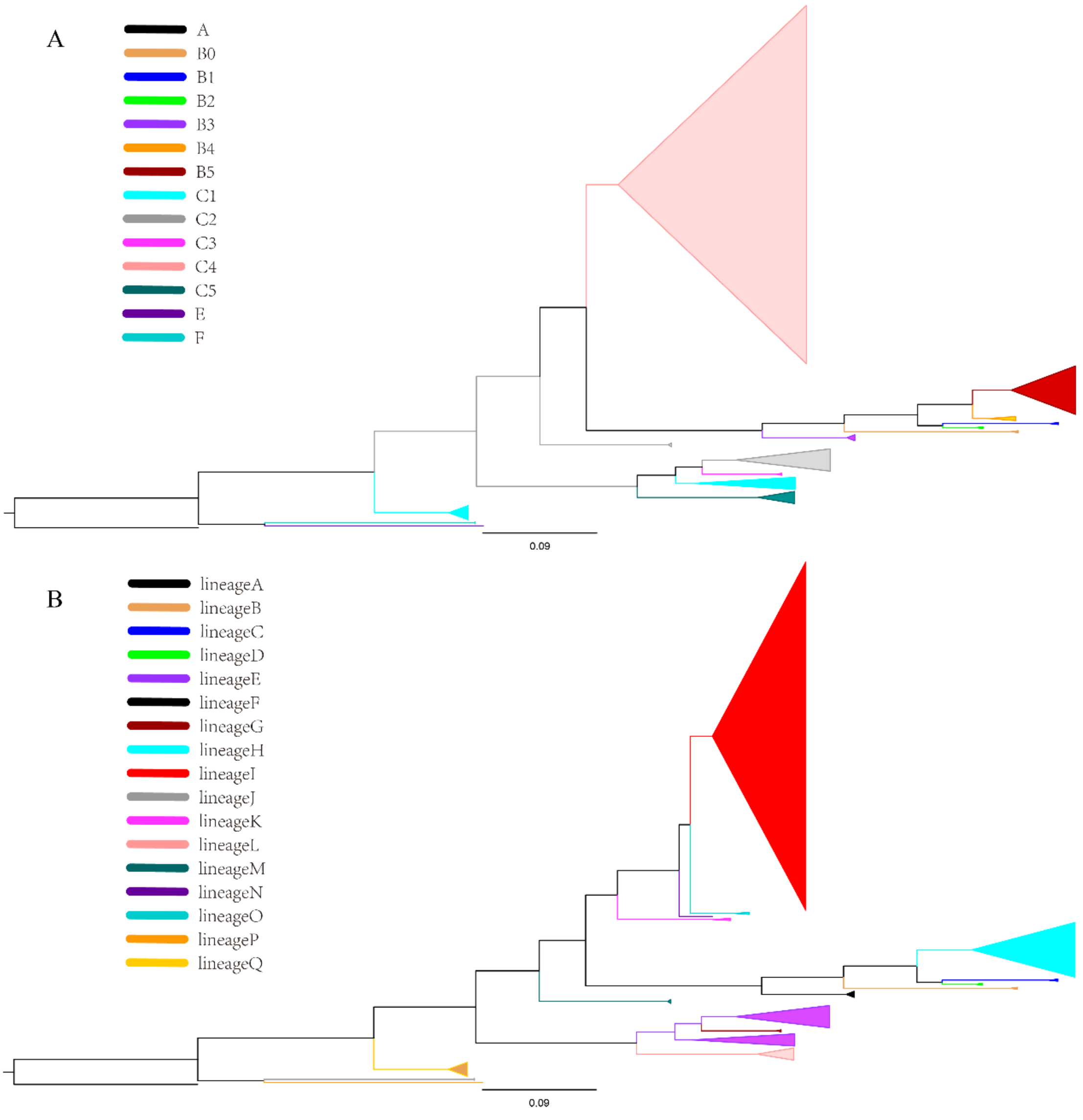

Fig. S5

Schematic diagram of sequence analysis. Schematic diagram of the sequence analysis and the corresponding datasets, different analysis modules with different colors.

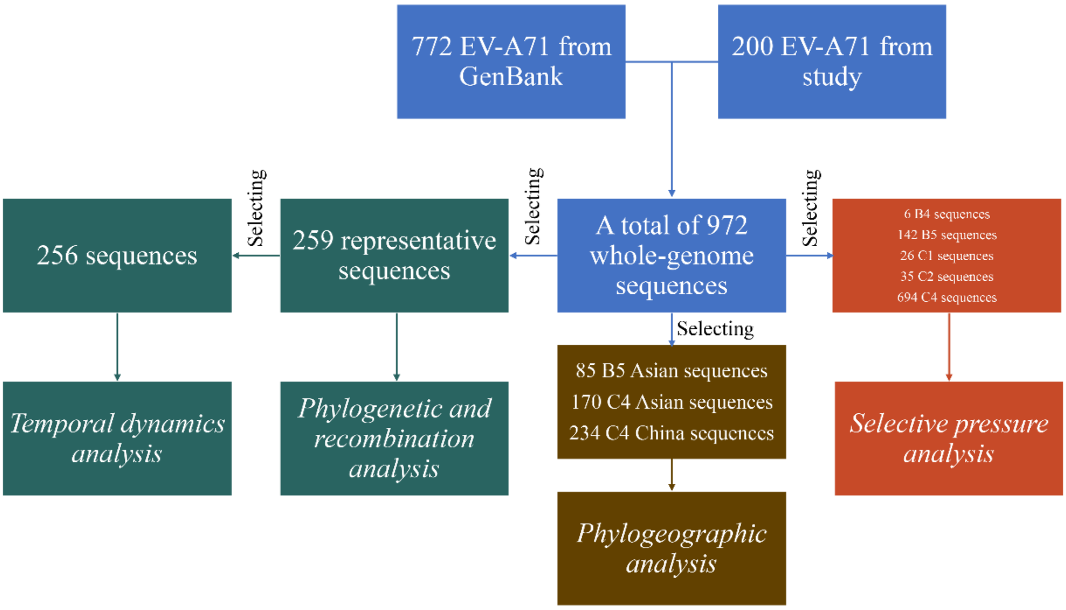

References

1. Yang CF, Naguib T, Yang SJ, Nasr E, Jorba J, Ahmed N, Campagnoli R, van der Avoort H, Shimizu H, Yoneyama T, Miyamura T, Pallansch M, Kew O. 2003. Circulation of endemic type 2 vaccine-derived poliovirus in Egypt from 1983 to 1993. J Virol 77:8366-77.
2. Duchêne S, Duchêne D, Holmes EC, Ho SY. 2015. The Performance of the Date-Randomization Test in Phylogenetic Analyses of Time-Structured Virus Data. Mol Biol Evol 32:1895-906.
3. Gao F, Liu X, Du Z, Hou H, Wang X, Wang F, Yang J. 2019. Bayesian phylodynamic analysis reveals the dispersal patterns of tobacco mosaic virus in China. Virology 528:110-117.
